# Non-randomised trial of a hepatitis C same-day test and treat model using antibody test only for people who inject drugs in Armenia, Georgia and Tanzania: a CUTTS HepC study protocol

**DOI:** 10.1136/bmjopen-2025-114119

**Published:** 2026-03-24

**Authors:** Bridget Louise Draper, Mia Flynn, Sophia Schroeder, Ernst Wisse, Faith Aikaeli, Zin Mar Han, Maureen Ayako, Sahar Bajis, Maia Butsashvili, Kamo Davtyan, Tea Kordzadze, Pauline Lamand, Niklas Luhmann, Knarik Sargsyan, Mbazi Senkoro, Nick Scott, Jack Stone, Peter Vickerman, Anita Voloshin, Josephine Walker, Annie Madden, Mark A Stoové, Margaret Hellard, Alisa Pedrana, on behalf of CUTTS HepC Consortium, Hepatitis C Models of Care Study Team, Ashot Davidyants

**Affiliations:** 1 Burnet Institute, Melbourne, Victoria, Australia; 2 Monash University, Melbourne, Victoria, Australia; 3 Médecins du Monde, Paris, France; 4 Department of Global HIV, World Health Organization, Geneva, Switzerland; 5 Georgia Health Research Union, Tbilisi, Georgia; 6 National Institute for Medical Research, Dar es Salaam, United Republic of Tanzania; 7 University of Bristol, Bristol, UK; 8 International Network of People who Use Drugs (INPUD), London, UK; 9 La Trobe University, Melbourne, Victoria, Australia; 10 Alfred Hospital, Melbourne, Victoria, Australia; 11 The University of Melbourne School of Population and Global Health, Melbourne, Victoria, Australia; 12 Doherty Institute, Melbourne, Victoria, Australia

**Keywords:** Hepatitis C, same-day treatment, low-and middle-income countries, Armenia, Georgia, Tanzania, point-of-care test, feasibility, effectiveness, acceptability

## Abstract

**Introduction:**

Despite the availability of curative treatments, hepatitis C diagnosis and treatment coverage is suboptimal globally with few countries on track to achieve the WHO’s 2030 elimination targets. In 2022, an estimated 50 million people were living with hepatitis C, with 1 million new infections annually. Most people living with hepatitis C reside in low- and middle-income countries, and people who inject drugs are disproportionately affected by hepatitis C.

Continuing simplification of diagnostic pathways and treatment care models is required to improve linkage to care and reduce costs associated with hepatitis C treatment and cure.

**Methods and analysis:**

This study is a multi-country non-randomised, quasi-experimental, prospective comparative two-arm trial. It aims to assess the feasibility of implementation, retention in hepatitis C care and achievement of cure and cost-effectiveness outcomes, comparing two simplified hepatitis C testing and treatment pathways.

Arm 1 is a standard simplified test and treat model of care following global guidance, and arm 2 is an innovative rapid, same-day treatment initiation model of care using a presumptive treatment approach based on shortened read-time of the point-of-care OraQuick hepatitis C antibody test result. Secondary outcomes include assessing the accuracy of the OraQuick hepatitis C antibody test in predicting viraemia and the acceptability of each pathway.

This study will be implemented in Armenia, Georgia and Tanzania. Treatment-naïve people who inject drugs aged over 18 years in each country will be eligible for enrolment.

Recruitment commenced in October 2024 in Armenia, June 2025 in Georgia and August 2025 in Tanzania and is anticipated to close by December 2026.

**Ethics and dissemination:**

This trial has been reviewed by WHO Ethics Review Committee (ERC), Alfred Hospital Ethics Committee (Australia) and local country ERCs. Alongside journal publications and conferences, the results from this study will be disseminated through summary reports and workshops with key stakeholders and with communities of people affected by HCV through relevant organisations/networks, including the global Community Advisory Board (CAB). The study results will inform national scale-up of simplified care models and inform potential pathways for further simplification of care models, including the potential for one-step diagnostic pathways and same-day treatment in particular scenarios for the three study countries, and other low- and middle-income countries globally.

**Trial registration number:**

NCT06159504.

**STRENGTHS AND LIMITATIONS OF THIS STUDY**

- Well-established connections and collaboration with local harm reduction networks through project design and implementation to align project methods and outcomes with community values, preferences and needs.
- Diverse implementation settings involving three countries at distinct phases in their hepatitis C response to demonstrate the effectiveness of a simplified model of care and utility of 5 min antibody read-time in different low- and middle-income country contexts.
- Detailed costing data for each model will enable robust cost-effectiveness outcomes and allow exploration of the impact of different models of care in different hepatitis C prevalence settings.
- There is a degree of uncertainty regarding the spontaneous clearance rate in each country, which will impact the proportion overtreated in arm 2; however, we have planned for a wide range (~0%–60%).
- This is a two-arm non-randomised prospective comparative trial, where arms 1 and 2 will be recruited consecutively. This may impact the recruitment rate and feasibility of reaching arm 2 treatment targets as well as the characteristics of participants being recruited; however, we will monitor recruitment and may re-allocate treatment targets from one country to another if necessary to reach required sample size.

## Introduction

### Background and rationale

 Globally, in 2022 there were an estimated 50 million people living with hepatitis C^1^ and 1 million new infections annually.^1^ If left untreated, hepatitis C can lead to liver cirrhosis, and in some cases, hepatocellular carcinoma and death. Most people living with hepatitis C reside in low- and middle-income countries (LMICs).^2^ Globally, people who inject drugs are a key population affected by hepatitis C, accounting for up to two-fifths (equivalent of approximately 800,000) of all new hepatitis C infections annually.^1 3 4^ Transmission risk in this group is predominately due to the re-use of injecting equipment. Recognising the burden of disease globally and following the advent of highly effective and tolerable curative direct-acting antiviral (DAA) treatment, WHO set targets for the elimination of hepatitis C as a public health threat by 2030.^5^ This included diagnosing 90% of people with hepatitis C and treating 80% and a specific target for incidence reduction to 2 per 100 person-years among people who inject drugs.^6^

Globally, between 2015 and 2022, hepatitis C diagnosis coverage was 36% and treatment coverage was 20%.^1^ The coverage of testing and treatment for hepatitis C differs by region, with diagnosis and treatment coverage ranging from 13% and 3% in WHO African region to 29% and 9% in WHO European region, respectively.^1^ While many countries have made substantial progress towards elimination, only 11 of 45 high-income countries globally are estimated to be on track to achieve the elimination targets.^7 8^ LMICs in particular are falling behind, with most not reaching the level of testing and treatment necessary to meet the elimination targets. A combination of factors, including limited financing of hepatitis C elimination programmes, centralised diagnostic services and restrictions on which types of clinicians can prescribe, is contributing to the challenge of hepatitis C elimination.^9^ Specifically, hepatitis C diagnosis and treatment coverage for people who inject drugs differs substantially globally, with estimated proportions ever receiving an antibody test ranging from <5% to over 75%, with treatment uptake mostly between 20% and 50%.^8^

Globally, rapid diagnostic tests (RDTs) to detect hepatitis C antibodies are widely available and generally affordable. These tests are simple to use, allow decentralisation and use at the point of care (POC) and are relatively low cost. However, a confirmatory test—either an RNA (PCR) or core antigen test—is required to confirm current infection. These tests generally require substantial referral processes and laboratory infrastructure to process samples, typically needing highly skilled and trained laboratory technicians and high-quality laboratory equipment. They are more expensive, costing from US$10 to US$38 depending on the architecture or device used.^10^

Advances in PCR testing technology have led to the availability of near or at the POC closed systems, which require only basic laboratory infrastructure and trained healthcare workers. The Cepheid GeneXpert platform is the most widely available platform in LMICs. POC or near POC platforms can enable the scale-up and increased reach of testing for key populations, including people who inject drugs, by improving accessibility of testing. However, this approach requires significant financial resourcing by service providers for infrastructure and human resources, particularly as POC platforms often sit outside of the public healthcare system. The machines are often siloed within specific disease programmes (ie, within national tuberculosis programmes), making them generally inaccessible to other disease programmes. Also, each individual test cartridge costs approximately US$15–US$35 (depending on negotiated price)^1^ and in many countries, costs substantially more for the consumer, depending on the price negotiations at a country level, as well as individual level costs. As a consequence, these tests are unaffordable for many people.

The cost of DAA treatment was initially a barrier to many LMICs rolling out hepatitis C programmes, but a 12-week course of generic pan-genotypic DAAs is now available for US$60–US$84.^11^ Currently, key issues impacting access in LMICs are test affordability and accessibility, with many LMICs and individuals struggling to afford the diagnostic tests to identify their hepatitis C status.

Recent global guidelines have simplified treatment protocols due to the safety and effectiveness of DAAs, enabling greater scale-up of treatment.^12^ Despite this, many LMICs still have restrictions on who can access DAAs and who can prescribe DAAs.^13^ It is imperative that efforts continue to remove these restrictions. People who inject drugs often face additional barriers to accessing care, including those arising from socio-economic factors, structural factors and stigma/discrimination.^8^ While in many LMICs, free-of-charge antibody screening tests are offered through harm reduction programmes via fixed site or outreach drop-in centres, the high out-of-pocket costs of a confirmatory RNA test at these sites (if available) or the requirement to attend centralised secondary/tertiary hospitals to have RNA testing deter people from this critical step in the care pathway. These issues in getting an RNA test can result in delays in offering treatment and consequently, a high proportion of people can be lost to follow-up after receiving their initial screening hepatitis C antibody test.^14^ Antibody testing without a complete diagnosis (ie, an antigen or RNA test to confirm virus) limits opportunity to link people to care and reduces the cost-effectiveness of programmes globally.^15^ Simplification of the diagnostic and clinical pathway is critical. Timely treatment, particularly for people who inject drugs, can also reduce onward transmission risk and prevent progression of liver disease.^16^

Emerging evidence for simplified diagnostic pathways and models of care, including same-day treatment models, from LMICs demonstrates the feasibility, acceptability and effectiveness of these approaches to increase treatment uptake, including among people who inject drugs.^17^,^22^ ‘Same-day test and treat’ models that have been piloted in Egypt, Malaysia and India have all relied on POC or near-POC HCV viral load (VL) testing (either on-site GeneXpert HCV VL or off-site Trunat testing) prior to commencing DAAs and on-site/same-day off-site biochemical analysis for liver function tests and platelets, or on-site FibroScan for liver cirrhosis assessment.^18^,^20^ These approaches require extended attendance time for patients to initiate treatment and are likely difficult to replicate in many settings due to associated costs and infrastructure requirements. For one ‘same-day test and treat’ model, which was implemented in a residential rehabilitation facility where the population was not leaving the location of care, the extended wait time was not a barrier to care.^20^

Previous work has explored the potential simplification of testing pathways, using existing tests but using a shortened read-time of the POC OraQuick rapid hepatitis C antibody test to identify those with current infection.^23^ The OraQuick rapid hepatitis C antibody test is a WHO prequalified test (self-test and professional use) that is designed to be read at 20 min, with 100% sensitivity and 99.7% specificity.^24^ In a study by Smookler *et al*, using pooled clinical and real-world cohorts from Canada and Spain, none of the participants whose HCV antibody tests turned reactive only after the 5 min mark were found to be RNA positive. A positive test result at 5 min identified all patients with viraemia with 100% sensitivity (95% CI 98.4% to 100%) and the negative predictive value was 100% (95% CI 94.9% to 100%).^23^ These findings could help reduce reliance on costly RNA testing in low-resource settings, as individuals with a non-reactive antibody result at 5 min are highly unlikely to have an active infection.

Importantly, if a participant’s antibody test was positive in <5 min, it did not imply that all participants had virus present, with a positive predictive value of 62%.^23^ In particular, the accuracy of the shortened read-time of the OraQuick rapid hepatitis C antibody test to identify patients with viraemia is greatly reduced among those who had recently been treated and achieved sustained virological response (SVR).^23^ Therefore, excluding those previously treated improves the accuracy of the 5 min read-time result in detecting only patients with viraemia.

Considering these accuracy outcomes, there are two potential utilities of the proposed shortened read-time of OraQuick rapid hepatitis C antibody tests. First, by reducing the need for unnecessary reflexive RNA testing among those who return a non-reactive result by 5 min given the accuracy to exclude individuals without current infection. Second, and the focus of this study, is the potential utility of using the 5 min read-time result to identify those with current infection and initiate same-day presumptive treatment for hepatitis C. The key risk of presumptive treatment of people relates to treating people with hepatic decompensation or other significant comorbidities, prior to receipt of recommended laboratory investigations. This risk can be minimised by following clear protocols on medical history taking, including a physical examination for clinical signs and symptoms of underlying conditions that might be a contraindication. In addition, at the individual level, treating those without current infection may result in them experiencing side effects of DAAs, similar to those who are treated for their current infection.

**Figure 1:**
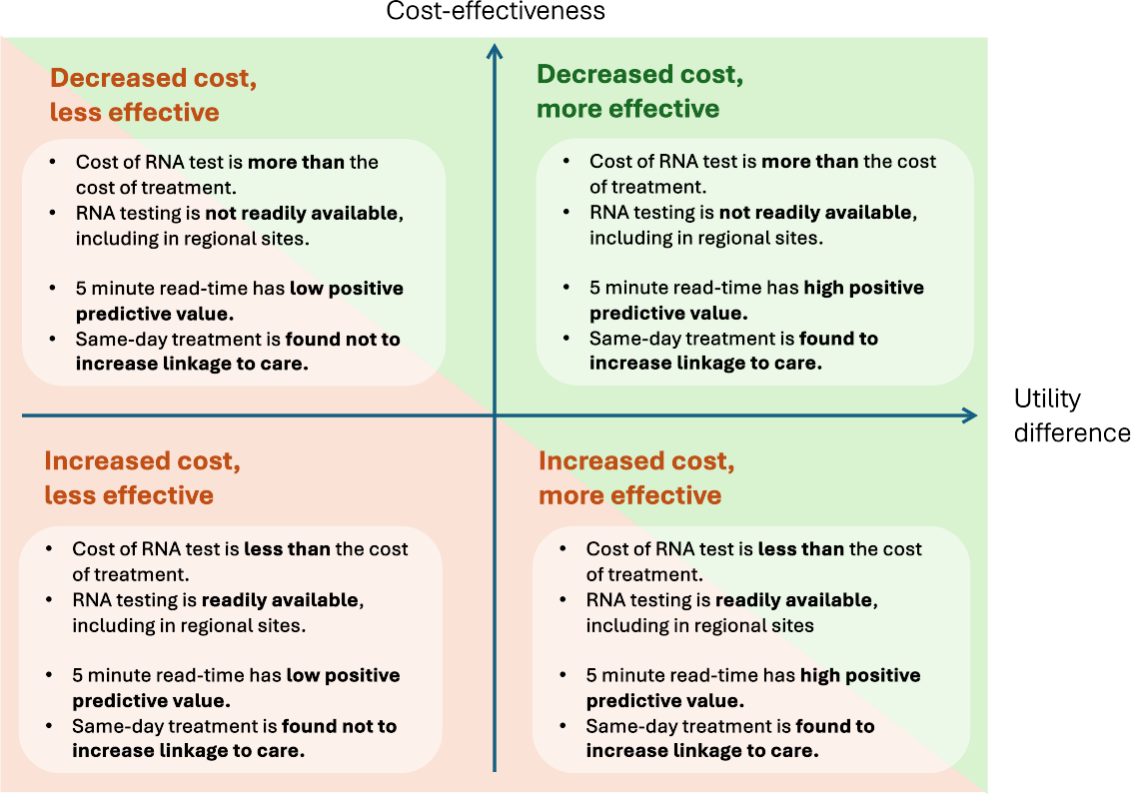
Exploring potential use-case scenarios for the 5 min read-time test to facilitate same-day treatment.

Models of care that seek to further simplify clinical pathways may reduce consultation times and reduce unnecessary costs of testing, enabling resources in LMIC to be better targeted and reduce wastage. The potential utility of this particular model depends on the balance of reducing testing costs and improving linkage to care against overtreatment, and the relative cost and availability of commodities and the underlying HCV prevalence in specific countries (figure 1). It is important to note that the acceptability of such an approach to both patients and providers, and to policy-makers, must also be established and will influence any future utility of this approach outside of a research setting.

### Trial study design

This paper describes the study protocol for a multicountry non-randomised, quasi-experimental, prospective comparative two-arm trial to assess the feasibility of implementation, retention in hepatitis C care and achievement of cure and cost-effectiveness outcomes, comparing two simplified hepatitis C testing and treatment pathways.

Arm 1 is a standardised simplified test and treat model of care following global guidance, and arm 2 is an innovative rapid, same-day treatment initiation using a presumptive treatment approach based on shortened read-time of the POC OraQuick hepatitis C antibody test result.^23^

Responding to WHO’s call for further simplification and decentralisation of hepatitis C testing and treatment, this study will be conducted in three LMICs, the Republic of Armenia (here, Armenia), Georgia and United Republic of Tanzania (herein, Tanzania), providing much-needed evidence from LMIC settings to increase access to hepatitis C treatment. In particular, this study is exploring opportunities to pursue a simplified and, ideally, one-step diagnostic algorithm, which is broadly recognised as a potentially major game-changer in HCV care.

All three countries have varied hepatitis C and injecting drug use epidemiology, harm reduction programming and national responses to hepatitis C. Georgia has a national decentralised hepatitis C programme, Armenia has a centralised programme only available in Yerevan, Tanzania has yet to implement a national programme. Given all three countries are in a distinct phase of their national response to hepatitis C, implementing the trial in these environments will provide insights into its feasibility and utility in each setting and allow for greater applicability of findings globally.

Participants will be consecutively enrolled into arm 1 (standardised simplified model of care), until the study’s country-level treatment initiation target is met. Then, participants will be consecutively enrolled into arm 2 (same-day treatment initiation), until the study’s country-level treatment initiation target has been met. Enrolment will close when arm 2 reaches country-level treatment initiation targets.

### Aims and research questions

This study aims to demonstrate and compare the feasibility, retention and cure rates between arm 1 and arm 2 and assess their cost-effectiveness.

The three primary research questions are:

1. Does the same-day treatment initiation model (arm 2) increase hepatitis C treatment uptake and achievement of SVR among people who inject drugs compared with the simplified care model (arm 1)?
2. What is the feasibility of implementing the hepatitis C simplified care (arm 1) and same-day treatment (arm 2) care models in the varied healthcare settings in the three study countries (Armenia, Georgia and Tanzania)?
3. What is the comparative cost-effectiveness between the same-day treatment initiation model (arm 2) and the simplified care model (arm 1)?

Secondary research questions include assessing participant acceptability of the two care models, assessing healthcare practitioner perspectives on the feasibility and acceptability of the care models and identifying barriers and enablers to support scale-up.

Additional secondary outcomes include the time to initiate treatment, proportion completing treatment in arm 1 and arm 2, the sensitivity and specificity of the 5 min read-time result, the optimal ‘cut-off’ read-time to accurately identify participants with viraemia and the proportion ‘overtreated’ for hepatitis C in arm 2. Feasibility of both care models (primary research question #2) will also be assessed through comparison of care cascade progression outcomes with available historical data, where feasible, and through an embedded mixed-methods process evaluation.

## Methods and analysis: participants, interventions and outcomes

### Study setting

#### Project countries

##### Armenia

Armenia is a small middle-income country in the South Caucasus region bordering Georgia, Turkey, Azerbaijan and Iran, with a population of 2.9 million.^25^

Armenia has a mixed hepatitis C epidemic, with general population hepatitis C antibody prevalence of 2%^26^ and higher prevalence of 39%^27^ among the estimated 14 000 people who inject drugs.^27^

The Armenian government has invested in hepatitis C by establishing a national hepatitis C programme, which provides HCV RNA testing and treatment (sofosbuvir/velpatasvir (SOF/VEL)) free of charge.^28^,^30^ The national programme is centralised at the National Center for Infectious Diseases (NCID) in the capital city, Yerevan, and follows a simplified clinical pathway, requiring specialist clinicians to prescribe treatment.^28^ The Ministry of Health is supportive of the decentralisation of care and task-shifting to general physicians, using this study as a pilot.

This study will provide the first evidence for the feasibility, effectiveness and cost-effectiveness of scale-up of treatment through a decentralised model, which provides DAAs outside Yerevan and through collaboration with harm reduction programmes.

##### Georgia

Georgia is a small middle-income country in the South Caucasus region bordering Russia, Turkey, Armenia and Azerbaijan, with a population of 3.7 million.^31^

Historically, Georgia had one of the highest reported prevalence estimates globally for hepatitis C among the general population, with 7.7% antibody prevalence and 5.4% RNA prevalence in 2015, corresponding to an estimated 130 000 chronic hepatitis C cases.^32^ In 2015, Georgia launched one of the first national elimination programmes globally, initially supported by a donation of DAAs from Gilead Sciences. This highly successful programme commenced with four sites in the capital Tbilisi and expanded to over 30 sites across the country by 2018^33^ and achieved high linkage to care.^34^ A recent sero-survey in 2021 shows a 67% reduction in RNA prevalence, down to 1.8% among the general population.^32^

Current estimates suggest hepatitis C viraemic prevalence remains high at 18%^32^ among the estimated 51 000 people who inject drugs.^35^ The national elimination programme predominately delivers testing and treatment through specialised treatment centres and allows for hepatitis C testing and treatment within selected harm reduction sites. However, on-treatment ALT monitoring was still required until 2025 and referral for those with FIB-4 score >1.45 to a specialist is still required by the national programme.

This study will address previous programme concerns around how the national programme can further scale up testing and treatment in the regions, and link people who inject drugs to timely treatment at harm reduction sites. The preparatory work for this study led to the removal of on-treatment laboratory monitoring for those on regimens without ribavirin. This study will contribute to the evidence of the safety of management of patients at harm reduction sites following a simplified care model, with specialist support as required.

##### Tanzania

Tanzania is a lower middle-income country in East Africa, with a population of 66.6 million.^36^ Tanzania borders seven countries, including Kenya, Rwanda and Mozambique.

In Tanzania, there is a concentrated epidemic of hepatitis C among people who inject drugs, with estimated antibody prevalence ranging from 30% to 75%.^37^,^40^ The general population antibody prevalence is estimated at 1%; however, this estimate is only based on blood donor prevalence studies and there has not been a nationwide sero-prevalence survey.^41^ There are an estimated 30 000 people who inject drugs in Tanzania,^42^ with most residing in Dar es Salaam and Zanzibar.^43 44^ Access to hepatitis C testing and treatment in Tanzania is extremely limited, population-level diagnosis coverage is reported to be 7% and treatment coverage 0% at the end of 2022.^1^

The Tanzanian government has identified ensuring accessible, reliable and affordable screening, diagnostic, care and treatment services as a key objective of its National Strategic Plan.^45^

Currently, testing for hepatitis C antibody is available through harm reduction programmes on an ad hoc basis, at enrolment into the medication-assisted opioid treatment (methadone) programme and via the newly established national programme based out of national hospitals (Muhimbili National Hospital and Mloganzila hospital), zonal hospitals, regional referral hospitals and all opioid agonist treatment (OAT) clinics. The Tanzania national programme was initiated mid-2025 and offers HCV antibody testing and treatment at no cost to patients. RNA testing requires an out-of-pocket cost of approximately US$70. Prior to this, RNA testing and treatment was only available through specialist physicians (hepatologists) at the Muhimbili National Hospital and private hospitals, incurring high out-of-pocket costs, with confirmatory RNA testing typically performed in neighbouring countries.

This study will provide the first evidence on how to cost-effectively scale up testing and treatment for key populations, including through integration into the existing OAT programme, decentralising care to a community-based centre and task-shifting care from exclusively hepatologists to a lower cadre of healthcare worker.

### Study sites

Study sites have been identified through consultation with Médecins du Monde, government, community, clinical and research networks in-country. Table 1 provides details of selected study sites and their functions.

**Table 1:** Study sites

Country	City/Region	Study site	Type of site
Armenia	Yerevan	Real World Real People*	Fixed site community-based organisation, with outreach NSP workers referring participants into clinical sites
	Yerevan	National Center for Infectious Diseases Medical Center	Secondary public hospital
	Syunik	Kapan Medical Center	Regional secondary public hospital
	Lori	Vanadzor Medical Center	Regional secondary public hospital
	Shirak	Gyumri Medical Center	Regional secondary public hospital
Georgia	Tbilisi	Hepa Plus	Fixed site community-based organisation, with outreach NSP
	Tbilisi/Rustavi	New Vector	Fixed site community-based organisation, with outreach NSP
	Gori	Step to Future	Fixed site community-based organisation, with outreach NSP
	Zugdidi	Xenon	Fixed site community-based organisation, with outreach NSP
	Batumi	Imedi*	Fixed site community-based organisation, with outreach NSP
Tanzania	Dar es Salaam	Mukikute	Fixed site community-based organisation, with outreach NSP
	Dar es Salaam	Temeke Regional Referral Hospital—Methadone Medication Assisted Treatment Clinic	Secondary public hospital

* This site is not a clinical site. The site does not enrol participants, only screens for eligibility and refers them to medical centres.

NSP, needle syringe programme.

### Patient and public involvement

#### Community involvement

This study is part of the Unitaid HCV Portfolio, which funded three Consortia to design and deliver implementation and research activities that contribute to closing the HCV prevention and testing gap through increased access to new or underused tools for prevention and enhanced simplified testing and treatment protocols for people who inject drugs in LMICs.

As part of the HCV Portfolio, the International Network of People who Use Drugs (INPUD) and Frontline AIDS convened a global Community Advisory Board (CAB) consisting of people with lived experience of injecting drug use with representation from all three Consortia’s implementing countries. The CAB members representing Armenia, Georgia and Tanzania were consulted for this study protocol development and will be involved in contributing to interpretation and dissemination of findings. Consultation throughout protocol development included review of participant facing materials such as participant information and consent forms, recruitment forms and education materials. Research questions and outcome measures were developed prior to consultation with CAB; however, the CAB provided positive feedback on the intended study design. Feedback from the CAB was considered for refining the conduct of the study and recruitment strategies.

Trained community researchers will be involved in conducting qualitative interviews on acceptability with trial participants where possible. In some settings, peer workers are involved in recruiting participants.

Regular CAB meetings and ad hoc consultation with country-specific CAB members and community networks will be held throughout the duration of the study. This will provide an opportunity for the community to be informed of study progress, inform interpretation of results by providing further contextual information and lived experience perspectives and consider the most appropriate dissemination strategies.

### Recruitment

Within each country, participating sites can have different recruitment functions including simply promoting the study and referring clients to a central service for recruitment or being a community-based recruitment site. The study will be promoted through promotional posters and flyers at the study sites and by outreach workers from study sites who will distribute promotional materials and discuss the study with potential participants, and provide facilitated referral into the study site in Armenia.

### Participant eligibility

Interested individuals will be screened for eligibility prior to referral to a clinical service.

Study inclusion criteria for participants are as follows:

- Aged 18 years or older.
- Be attending a listed referral needle/syringe programme site or self-report ever injecting drugs.
- Must not be currently on or previously had treatment for hepatitis C.
- Be able and willing to provide informed consent in the local language.
- Be a citizen of the country (Armenia and Georgia—citizenship is not an inclusion criteria for Tanzania).
- Self-report not living with HIV (Georgia only).

Exclusion criteria are as follows:

- Women known to be currently pregnant or who are breastfeeding.
- Individuals currently engaged in hepatitis C treatment.
- Unable to provide informed consent.
- Individuals diagnosed with a current hepatitis B infection or uncontrolled/newly diagnosed HIV infection (HIV controlled infection defined as clinically stable and on treatment for at least 12 months).
- Individuals with suspected tuberculosis infection or on preventive tuberculosis therapy (Tanzania only).
- Individuals with evidence of decompensated cirrhosis of the liver.
- Individuals with other significant comorbidity that requires specialist management of hepatitis C treatment (eg, renal dysfunction).
- Unable to cease taking contraindicated medicines for DAAs.

All participants will be given details of the study via the patient information sheet and be required to provide written consent prior to enrolling in the study. The participant information sheet is provided in local language (Armenian, Georgian and Tanzanian Swahili) and will be verbally explained to potential participants by delegated study staff obtaining consent.

### Intervention

**Figure 2:**
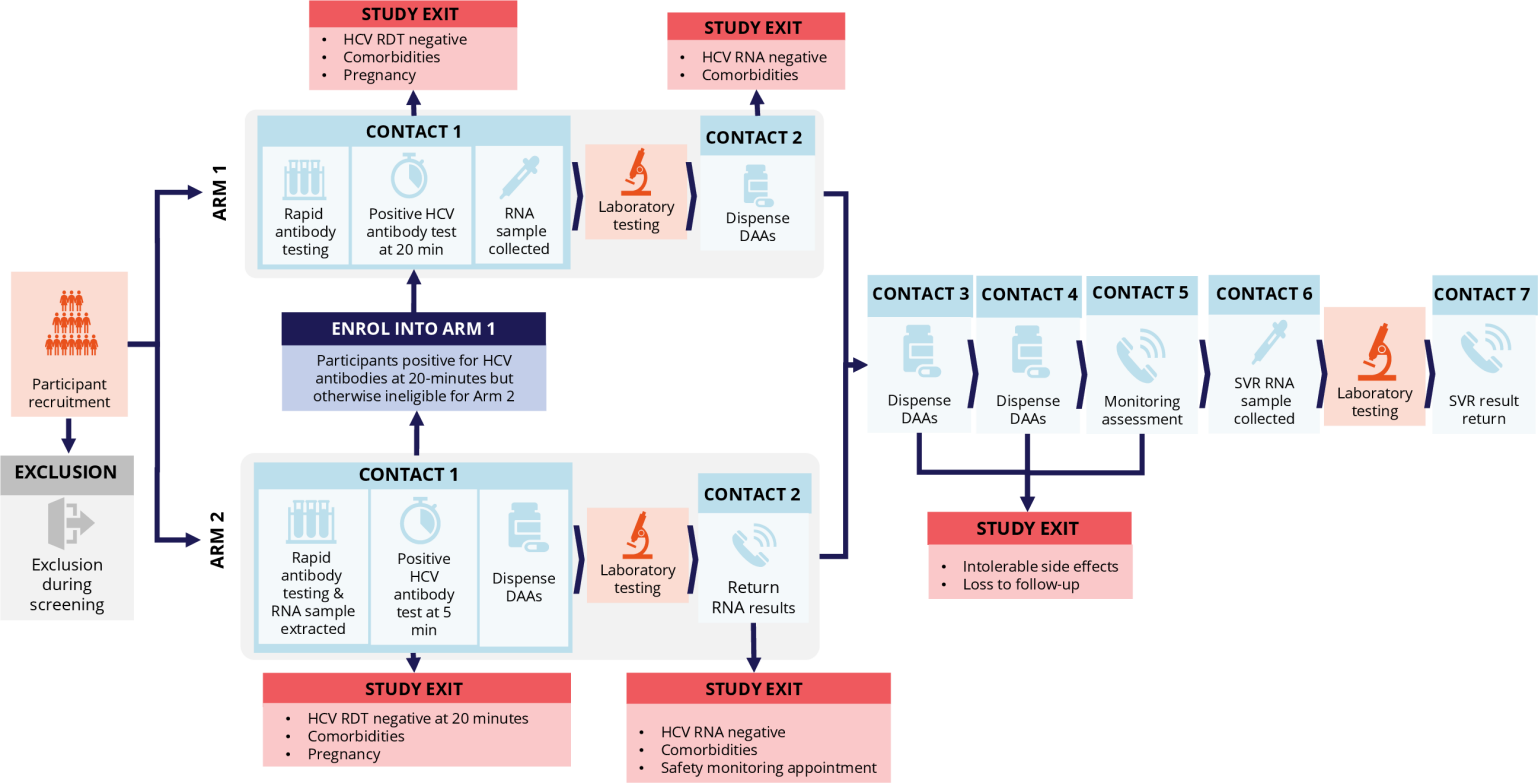
Arm 1 and arm 2 study procedures. DAA, direct-acting antiviral; HCV, hepatitis C virus; RDT, rapid diagnostic test; SVR, sustained virological response.

This study will implement two comparison intervention arms and no control arm, as shown in figure 2. Final recruitment, retention and outcome assessments will be presented in accordance with the Consolidated Standards of Reporting Trials (CONSORT) statement, including CONSORT diagrams. Arm 1 will implement a rapid POC antibody test, in addition to on-site venipuncture for confirmatory RNA testing and pretreatment workup investigations during the initial baseline visit; treatment initiation will occur at the second visit. Arm 2 will implement a rapid POC antibody test and same-day treatment for all participants who test antibody positive at 5 min at the baseline visit. They will have on-site venipuncture for confirmatory RNA testing and other clinical investigations, and the decision to continue or cease treatment based on RNA test results will occur at the second visit. All eligible participants will be offered SOF (400 mg)/VEL (100 mg), to be self-administered orally once per day for 12 weeks. All POC antibody tests will be sourced and funded by the study sponsor Médecins du Monde. The manufacturer differs by country; details are provided in online supplemental material 1.

All study data will be recorded in clinical case report forms (CRFs) specific to the visit.

#### Arm 1 study procedures

##### Visit 1: enrolment, laboratory investigations, physical examination and medical history taking

Eligible participants enrolled in the study will have a OraQuick rapid hepatitis C antibody test using a fingerstick specimen, according to the manufacturer’s instructions, with the result documented at 20 minutes. In addition, the OraQuick rapid test result for the first 5 min will be documented at 1 min intervals, including with a photograph to record whether the test is reactive or non-reactive at that time point. The exact time at which the test first turns reactive will also be recorded. The result at 20 min will be provided to the participant. If the antibody result is negative, confirmatory testing will not be offered and the participant will exit the study with no further investigations performed. If the antibody result is positive, blood samples will be collected for confirmatory laboratory RNA testing and additional pretreatment investigations including complete blood count (platelets, haemoglobin), liver function tests (alanine aminotransferase (ALT), aspartate aminotransferase (AST), albumin, bilirubin) and renal function tests (serum creatinine±estimated glomerular filtration rate (eGFR)). The FIB-4 score will be calculated and used to determine cirrhosis status.

In addition, additional rapid tests will be conducted at baseline visit:

- HIV antibody 1/2 RDT using venous blood sample.
- Hepatitis B surface antigen (HBsAg) RDT using venous blood sample.
- Urine dip-stick pregnancy test for all female participants with childbearing potential aged 18–49 years (pregnancy test can also be completed as part of pretreatment laboratory investigations in arm 1).

The brands of HIV and HBsAg RDTs will be included in online supplemental material 1; brands differ by country, depending on national procurement.

In Tanzania and Armenia, those who return a non-reactive HBsAg RDT and have no evidence of prior hepatitis B vaccination will be offered vaccination through the study. Evidence of vaccination is sourced through medical records accessible by study sites. In Georgia, those eligible for vaccination will be referred to a newly established national programme for vaccination.

Those newly diagnosed with HIV will be referred to treatment and be allowed to represent for study participation once their HIV is clinically stable, if recruitment is still open at that time in Armenia and Tanzania.

###### Physical examination, medical history and drug-drug interactions

Participants will undergo physical examination to exclude suspected decompensated cirrhosis (portal hypertension, variceal haemorrhage and hepatic encephalopathy, coagulopathy or liver insufficiency, palmar erythema, nasal bleeding and oedema). Participants will have their medical history taken and current medications/supplements recorded.

Any participants with suspected decompensated cirrhosis or other comorbidity that may exclude the participant from this study will be referred to a specialist for further review and care.

Drug-drug interactions with SOF/VEL will be assessed using the Liverpool Drug Interactions online resource (Liverpool HEP Interactions) and any amber/red drug-drug interactions must be resolved with input from the participant and other treating/prescribing clinician(s) to assess appropriateness of ceasing/altering these treatments before DAA can be initiated.

The baseline participant questionnaire will be administered by study staff. This questionnaire collects information on demographic characteristics, history of incarceration, injecting practices, employment, income and productivity, healthcare utilisation, costs of attending for hepatitis C care and quality of life (EuroQol 5-Dimension 5-Level questionnaire(EQ-5D-5L) and EuroQol Visual Analogue Scale (EQ-VAS)).

### Visit 2: confirmatory laboratory results and treatment plan

Following receipt of the HCV RNA test result:

- If the confirmatory laboratory HCV RNA result is negative, the study nurse/doctor will communicate this to the participant (via phone call or in person). The participant will exit the study here. Standard harm reduction messaging and window periods for detection of HCV will be discussed with the participant.
- If the confirmatory laboratory HCV RNA result is positive, the participant will be assessed for treatment eligibility based on other laboratory investigations.
- For assessment of liver cirrhosis, participant pathways are outlined per country in table 2.

**Table 2:** Differentiated care pathways by Fibrosis-4 index (FIB-4) score result

FIB-4 score	Armenia	Georgia	Tanzania
<1.25	A FIB-4 score <1.25 indicates the individual is unlikely to have cirrhosis of the liver. Participants with a FIB-4 score <1.25 will be eligible to be prescribed SOF/VEL without further assessments.		
1.25–2.67	Participants will continue to start DAAs but can be referred for specialist review at the clinician’s discretion.	Participants with FIB-4 scores of 1.25–1.45 are considered to have ‘low fibrosis’ and will continue to start DAAs without further assessments at the study site. Participants with a FIB-4 score of >1.45–3.25 require FibroScan and review by a consulting specialist through case review of investigations with the study doctor, and if required, in-person appointment.	Participants will continue to start DAAs but can be referred for specialist review at the clinician’s discretion, in particular for ongoing monitoring.
>2.67–3.25	Participants will be reviewed by the supervising infectious diseases specialist and a decision about referral to a hepatologist will be made together with the treating study clinician.		
≥3.25	Participants with a FIB-4 score >3.25 must be referred to a hepatologist/gastroenterologist for further assessments prior to initiating treatment, given the higher risk of decompensated cirrhosis.		

DAA, direct-acting antiviral; SOF/Vel, sofosbuvir/velpatasvir.

In addition, the participant will be referred to a hepatologist/gastroenterologist if the participant’s laboratory results indicate any of the specified additional risk factors for decompensated cirrhosis (ALT or AST >200 U/L, bilirubin >1.14 mg/dL, albumin <3.5 g/dL without other obvious cause, or past/current jaundice, ascites, hepatic encephalopathy or haematemesis and melena).

If the specialist determines that the participant does not have decompensated cirrhosis, then they can receive DAA treatment as part of the study in arm 1 and will be followed up according to study timelines.

If the participant has elevated serum creatinine, the study doctor should calculate the eGFR. If eGFR is <30 mL/min/1.73 m^2^, they will be excluded from receiving DAA therapy as part of the study and referred for specialist review for ongoing care.

If the participant is deemed eligible for treatment, the study doctor will provide them with the first 4 weeks of SOF/VEL (28-day bottle) and ask them to begin treatment.

### Visit 3: treatment monitoring and dispensing

- Participants will attend the study clinic between 0 and 7 days prior to week 4 of their treatment plan to collect the next 4 weeks (28-day bottle) or remaining 8 weeks (56-day bottle) of treatment, with number of weeks dispensed at clinician’s discretion.
  - In Georgia, only 4 weeks of treatment will be dispensed at a time (as per national programme protocol).
  - In Tanzania, 4 weeks of treatment will be dispensed routinely; however, 8 weeks of treatment will only be dispensed when the participant requests it and the clinician approves necessity.
- Participants’ self-reported adherence to treatment will be assessed. Any side effects and any adverse events will be documented.
- In the event of missed doses, additional weeks of treatment may be provided at the clinician’s discretion, in consultation with the Principal Investigator.

### Additional on-treatment monitoring and dispensing visits

- For participants in Georgia, participants will attend the study clinic when they are due for the next 4 weeks of treatment to collect the next bottle. In addition, participants will receive a week 12 monitoring appointment.
- For participants in Tanzania, most participants will attend the study clinic between 3 and 7 days prior to week 8 of their treatment plan to collect the final 4 weeks (28-day bottle) of treatment at visit 4. In addition, participants will receive a week 12 monitoring appointment.
- For participants in Armenia, there will be no scheduled in-person appointment or laboratory monitoring during treatment between weeks 4 and 12, unless scheduled at the clinician’s discretion. Participants will receive a scheduled telephone call or in-person appointment at weeks 8–12 to assess treatment adherence and side effects, and to address any issues with lost medication.

### Treatment outcome assessment (SVR weeks 4–20)

- Participants will attend the study clinic to have venous blood samples taken for RNA testing to confirm if they have achieved SVR post-treatment.
- Participants can be assessed for SVR anytime from ≥4 weeks post-treatment completion until 20 weeks post-treatment completion*.
- Participants will complete the interviewer-administered SVR participant questionnaire, covering similar domains to the baseline questionnaire.

SVR will be defined as ‘HCV VL <10 IU/mL’ or ‘HCV VL Not Detected’, if using GeneXpert HCV VL test/other quantitative test, and as ‘HCV RNA not detected’, as per laboratory procedures if using another HCV RNA test.

*In secondary analyses, if an RNA negative result is returned after 20 weeks, it will be categorised as achieving SVR. If a positive result is returned after 20 weeks, the participant will be categorised as ‘indeterminate SVR’, given the uncertainty regarding whether treatment was unsuccessful or the participant had been re-infected with hepatitis C.

### Study exit

Participants may exit the study at various time points: when they choose to withdraw from the study, when the clinician identifies a medical issue/ineligibility prompting participant withdrawal or when they have been identified as lost to follow-up due to no longer being contactable.

Throughout, there will be a minimum of three attempts over up to 3 weeks to contact participants to arrange the follow-up appointment if the participant misses the scheduled appointment. In the case of SVR, participants will remain in the study until the SVR20 time point passes; they will be contacted up to four times, every 4 weeks to attempt to arrange for SVR appointment.

### Arm 2 study procedures

#### Visit 1: enrolment, laboratory investigations, physical examination, medical history taking, drug-drug interactions review and presumptive treatment prescription

Eligible participants enrolled in arm 2 will undergo an OraQuick rapid hepatitis C antibody test using a fingerstick specimen as per arm 1. The result at 5 min will be provided to the participant as a preliminary result and used to inform whether same-day treatment is offered. This is the key difference between arm 1 and arm 2, with participants in arm 2 offered presumptive treatment based on this result at 5 min, without waiting for the RNA result.

Laboratory testing on a venous sample is then required to confirm whether the participant has current infection. If the antibody result is negative, confirmatory testing will not be offered and the participant will exit the study with no further investigations performed (as per arm 1).

If the antibody result is positive, blood samples will be collected for confirmatory laboratory RNA testing and additional investigations (as per arm 1). The FIB-4 score will be calculated and used to determine cirrhosis status.

- If the POC result is positive at 5 min, blood samples will be collected for confirmatory laboratory RNA testing and additional investigations. Additional rapid tests, including HIV antibody 1/2 RDT, HBsAg RDT, rapid urine pregnancy tests, physical examination for signs and symptoms of decompensated cirrhosis, medical history taking and drug-drug interactions chart review for contraindications for same-day treatment will be conducted. Any participants with suspected decompensated cirrhosis or other comorbidity that may exclude participants from treatment in arm 2 of this study will be referred to a specialist for further review and care.
- If the POC result is a negative antibody test at 5 min and a positive test at 20 min, blood samples will be collected for confirmatory laboratory RNA testing and additional investigations, and the participant will be told that their test indicates they have been exposed to hepatitis C but may have a current infection. These participants are ineligible for same-day treatment in arm 2, but if their RNA test is subsequently positive, they will be offered treatment as per study procedures of arm 1 of the study but will not count towards the enrolment target.

Participants will be eligible for same-day treatment if they have:

- No physical signs of decompensated cirrhosis.
- No comorbid conditions that require further investigation or referral to a specialist.
- No contraindicated medications (amber/red using online Liverpool Drug Interaction Checker).

If the participant is deemed eligible for treatment, the study doctor will provide 28 days of SOF/VEL and the participant will be asked to begin treatment. The blood sample for confirmatory RNA testing must be collected prior to providing any treatment medication. If there is an issue with the blood sample itself, the participant will be asked to return to the study site as soon as possible for a follow-up blood draw. Only after confirmatory RNA laboratory results will a second bottle of 28 SOF/VEL doses be provided for the participant to continue their treatment course.

#### Visit 2: confirmatory laboratory results and treatment plan

- If the confirmatory laboratory HCV RNA result is negative, the study nurse/doctor will contact the participant (via phone call immediately) and the participant will be asked to stop treatment immediately and to return unused DAAs either directly to the clinic or an outreach worker, or in some cases via a prepaid envelope provided to them in advance. This group of participants will be actively followed up for a face-to-face appointment to ensure they understand the test result and implications and to collect information on their experience of treatment. However, following this next visit, the participant will exit the study. Standard harm reduction messaging and window periods for detection of HCV will be discussed with the participant.
- If the confirmatory laboratory HCV RNA result is positive, the participant will be assessed for eligibility for treatment continuation based on other laboratory investigations. If a participant is eligible, the participant will continue treatment for a total of 12 weeks.

Participants returning laboratory investigations indicating renal dysfunction, with abnormally high levels of serum creatinine where eGFR <30, will be asked to pause treatment while they seek specialist review and advice on whether to continue treatment.

Participants returning laboratory investigations indicating cirrhosis (FIB-4 score >3.25; ALT or AST >200 U/L; bilirubin >1.14 mg/dL; albumin <3.5 g/dL without other obvious cause) will be discussed with supervising specialist for a decision regarding pausing treatment or organising a specialist referral for review without pausing treatment.

#### Treatment monitoring, dispensing and outcome assessment

Once HCV RNA status is confirmed, follow-up will continue as per arm 1 study procedures.

### Study outcomes

The three primary research questions and outcomes are:

1. Does the same-day treatment initiation model (arm 2) increase hepatitis C treatment uptake and achievement of SVR (cure) among people who inject drugs compared with the simplified care model (arm 1)?
2. What is the feasibility of implementing a decentralised hepatitis C simplified care model (arm 1) and the same-day treatment care model (arm 2) in the varied healthcare settings in the three study countries (Armenia, Georgia and Tanzania)?
3. What is the comparative cost-effectiveness between the same-day treatment initiation model (arm 2) and the simplified care model (arm 1)?

The secondary research questions and outcomes pertain to the acceptability of the two care models to participants, to healthcare practitioners including the barriers and enablers to support scale-up of these models in country. Additional secondary outcomes include:

- the time to treatment initiation and the proportion completing treatment;
- the test performance (sensitivity, specificity, positive predictive values and negative predictive values) of the 5 min read-time OraQuick HCV antibody result in identifying participants with viraemia from those without viraemia;
- the optimal ‘cut-off’ read-time to identify participants with viraemia (using arm 1 and arm 2 data) to reduce the proportion of participants without viraemia who are ‘overtreated’ for hepatitis C in the same-day treatment initiation model of care;
- comparison of the care cascade for both arms 1 and 2 with available historical data, where feasible, including the proportion of people who inject drugs reached through the models.

### Participant timeline

Online supplemental material 5 displays the participant timeline.

### Sample size

The primary study aim (aim 1) is to assess if same-day treatment initiation model (arm 2) increases hepatitis C treatment uptake and achievement of SVR, compared with a simplified care model (arm 1). The study is powered to measure the primary outcomes of treatment uptake and cure in arm 1 and arm 2 in a pooled analysis of all three countries. In addition, we will compare retention in care between arm 1 and arm 2, in each country individually. The sample size is presented below (table 3).

**Table 3:** Country and site enrolment and treatment targets

Country	Site	Target enrolment	Target DAA initiation arm 1	Target DAA initiation arm 2
Armenia	Yerevan, National Center for Infectious Diseases	500	58	58
	Shirak, Gyumri Medical Center	164	19	19
	Lori, Vanadzor Medical Center	163	19	19
	Syunik, Kapan Medical Center	163	19	19
	**Total (Armenia**)	**990**	**115**	**115**
Georgia	Tbilisi, Hepa plus	1100	18	18
	Rustavi, New Vector	360	12	12
	Gori, Step to Future	440	42	42
	Xenon	1200	48	48
	**Total (Georgia**)	**3100**	**120**	**120**
Tanzania	Dar es Salaam, Mukikute	522	60	60
	Dar es Salaam, Temeke Regional Referral Hospital MAT Clinic	478	55	55
	**Total (Tanzania**)	**1000**	**115**	**115**
**Total**		**5090**	**350**	**350**

* Sample sizes based on the following antibody and RNA prevalence estimates: Armenia: 39% HCV Ab prevalence, 75% HCV RNA prevalence. Georgia: 58% HCV Ab prevalence, 30% HCV RNA prevalence. Tanzania: 40% HCV Ab prevalence, 75% HCV RNA prevalence.

Ab, antibody; DAA, direct-acting antiviral; HCV, hepatitis C virus.

#### Sample size calculation

We have considered two study designs to inform sample size calculations for the intervention arms.

#### Superiority of ‘same-day initiation’ (arm 2) versus ‘simplified care pathway’ (arm 1)

Acknowledging there are considerable uncertainties around expected uptake of DAAs in both arms, we examined the required sample size in various scenarios of treatment uptake where the intervention (arm 2) would be 5%–10% superior to the simplified care (arm 1). A sample of 700 (350 in each arm) would enable a minimum effect size of 7% favouring arm 2 (presuming 85% uptake in arm 1 simplified care arm) to be detected with 80% power at the 5% significance level. We have assumed 85% treatment initiation based on previous studies that have implemented simplified care pathways in LMIC settings,^17^ accounting for a 15% loss to follow-up prior to treatment initiation. We expect cure rates to be equal across both arms (~95%).

#### Equivalence of ‘same-day initiation’ (arm 2) versus ‘simplified care pathway’ (arm 1)

In the event that we cannot demonstrate superiority of the intervention (arm 2), we have sufficient power with a sample of 700 (350 in each arm) with 1:1 allocation to demonstrate equivalence of the two arms (with equivalence level set at 10%) if uptake in both arms ranges from 75% to 95%.

### Statistical analysis

Primary research question #1: Does the same-day treatment initiation model (arm 2) increase hepatitis C treatment uptake and achievement of SVR (cure) among people who inject drugs compared with the simplified care model (arm 1)?

#### Assessing outcome 1: impact of same-day treatment initiation model on treatment uptake and cure (arm 2 vs arm 1)

The proportion of participants initiating hepatitis C treatment and cured in the simplified care model (arm 1) compared with the same-day treatment care model (arm 2) will be calculated.

The outcome of treatment uptake will be a binary outcome measured by the proportion of participants who are RNA positive and are prescribed treatment and who self-report initiating treatment. The outcome of cure will be measured by the proportion who achieve SRV among those prescribed treatment. Analyses will be conducted using a per-protocol basis, with patients analysed according to the appropriate treatment allocation and study arm following eligibility and exclusion study criteria. Those who are enrolled in arm 2 and later treated via arm 1 study procedures are excluded from primary analyses.

All available binary outcomes will be analysed at the patient level using mixed-effects logistic regression models with random intercepts for clinic and fixed effects for interventions. Models will adjust for covariates, based on any differences in participant characteristics between arm 1 and arm 2 at baseline. These covariates may include: opioid substitution therapy status, injecting frequency, history of incarceration and housing status. ORs and 95% CIs will be reported.

Where data are available, treatment uptake and cure outcomes from arms 1 and 2 will also be compared with available historical data specific to each study country (Armenia, Georgia and Tanzania) and/or globally.

Primary research question #2: What is the feasibility of implementing a decentralised hepatitis C simplified care model (arm 1) and the same-day treatment care model (arm 2) in the varied healthcare settings in the three study countries (Armenia, Georgia and Tanzania)?

#### Assessing outcome 2: feasibility of simplified and same-day care models

A primary study aim (aim 2) is to assess and compare the feasibility of implementing a simplified care model versus same-day test and treat model within primary/secondary health services and harm reduction settings.

Feasibility of implementation will be assessed based on retention in care numbers and complemented by the effectiveness outcomes, including treatment uptake and SVR outcomes. Descriptive statistics will be used to calculate the proportion of participants progressing through the cascade of care, including proportion of participants who test antibody positive and then RNA positive for hepatitis C, and the proportion who then commence on treatment in the simplified model of care (arm 1) and in the same-day treatment model (arm 2).

In addition, feasibility and implementation requirements, and broader implementation and scalability considerations, will be assessed through a mixed-methods process evaluation (described below). The updated Consolidated Framework for Implementation Research (CFIR) by Damschroder *et al*^46^ and the recommended adaptations for LMICs by Means *et al*^47^ will be used to develop the data collection tools and analysis framework to guide the feasibility assessment. Data will be drawn from the process activity logs, monitoring visits and meetings, reflections and learning workshops and key informant interviews. For comparison across the sites, the implementation of the models of care at each site will be described following the Template for intervention description and replication (TIDieR) checklist and guide^48^ to provide a standardised description of study implementation.

This will contribute to an assessment of whether the model of care was implemented with fidelity and if effectiveness outcomes can be effectively replicated, providing evidence for the potential successful scale-up.

Primary research question #3: What is the comparative cost-effectiveness between the same-day treatment initiation model (arm 2) and the simplified care model (arm 1)?

#### Assessing outcome 3: comparative cost-effectiveness of same-day model versus simplified model (arm 2 vs arm 1)

A mathematical model will be used to estimate average hepatitis C-related costs and quality-adjusted life years per person for a theoretical cohort of 100 people in each country under three scenarios: (1) no treatment; (2) the simplified care model and (3) the same-day treatment model. Prevalence of hepatitis C antibodies and RNA in each country will be based on study data. The effect of the different models (arm 1 simplified care model and arm 2 same-day treatment model) on linkage to care and treatment uptake will be based on the pooled study outcomes across countries, and costs calculated using a decision tree structure capturing the cascade of care. The average country-specific cost per person in each scenario will be estimated from study data, using a health systems perspective (ie, consumables, medicines, health provider time and other costs identified by project staff as relevant for intervention delivery) and a societal perspective (ie, also including lost productivity among people with hepatitis C). For each country, health-related quality of life weights will be estimated using data from the EQ-5D-5L tool in each country. The incremental cost-effectiveness ratio (difference in cost divided by difference in quality-adjusted life years) will be calculated for the simplified care scenario compared with the no treatment scenario, and for the same-day treatment scenario compared with the simplified care scenario.

Estimates of average cost per person and treatment uptake for the simplified care and same-day treatment models (including the cost of the participants overtreated) in each country will be used as inputs to a national dynamic transmission model, which will be used to estimate the potential population-level benefits of scaling up each model of care.

#### Assessing secondary outcomes

##### Impact of same-day treatment initiation model on treatment completion and cure

Participants will be classified as completing treatment if they self-report that they have taken at least 8 weeks of treatment, with <14 days missed doses. Proportion completing treatment will be assessed among those who were confirmed RNA positive. The proportion of participants who complete hepatitis C treatment will be compared between arms. We will also assess treatment completion based on completion of the full 12-week treatment course.

The proportion of participants who achieve SVR (4–20 weeks post-treatment completion) among those who were confirmed RNA positive and initiated hepatitis C treatment in the simplified care model (arm 1) will be compared with the same-day treatment care model (arm 2), using per-protocol analysis of those allocated to each arm.

##### Time to initiate treatment

The time from HCV antibody RDT to prescription and dispensing of first 4 weeks of DAAs, and to self-report taking first tablet of treatment will be calculated, and compared across arms. These will be calculated both among those who were HCV antibody positive (arm 2) and also among those confirmed RNA positive (arms 1 and 2) and initiated hepatitis C treatment.

##### Accuracy of the shortened read-time of the HCV RDT

The test performance will be determined by calculating the sensitivity and specificity of the shortened 5 min read-time result, and the positive predictive values and negative predictive values. The proportion of participants with concordant results from the OraQuick rapid hepatitis C antibody test at the shortened 5 min read-time and from the confirmatory hepatitis C RNA test result will be calculated using data from both arms. Where data are available, we will also assess the performance characteristics of the rapid test by HIV status.

##### Optimal ‘cut-off’ for HCV RDT read-time

The time to positive HCV RDT under 5 min compared with RNA positivity will be assessed, including at 1 min intervals and the time turned positive.

##### Proportion ‘overtreated’ for hepatitis C

The proportion of participants ‘overtreated’/‘unnecessarily treated’ for hepatitis C in the same-day treatment model (those who are HCV antibody positive at 5 min but RNA negative), where participants were prescribed hepatitis C treatment or initiated hepatitis C treatment and later received a hepatitis C RNA negative test result will be calculated. The proportion of this group experiencing adverse events will also be described.

### Process evaluation: feasibility and implementation requirements

The feasibility and implementation requirements component of this study will involve a process evaluation of the implementation of the trial to understand the contextual factors influencing the trial outcomes and the scalability of these care models trialled in arm 1 and arm 2.

This component is a mixed-methods evaluation that will involve multiple data sources, including site assessments, interviews with study staff and other stakeholders and a process monitoring and activity log that captures challenges and resolutions.

A baseline site assessment and regular site monitoring assessments will capture information about the site, how the site operates and fidelity of study implementation. Semi-structured interviews will be conducted with study site staff (doctors, nurses) and laboratory staff involved in the study to understand their perspectives on implementing this study. The structured process monitoring and activity log will be completed regularly by the study coordination team in collaboration with country teams, capturing challenges and solutions arising during study implementation.

A reflection, learning and dissemination workshop will be conducted in each country towards study close to capture perspectives of various stakeholders on the preliminary study findings and potential for scalability.

Online supplemental material 2 provides an overview of the feasibility and implementation requirements assessment, mapped to the domains of the updated CFIR.

### Participant acceptability

A participant acceptability substudy will employ qualitative methods using in-depth, semi-structured interviews with participants of arm 1 and arm 2 to examine their experiences of, perceptions and attitudes about accessing hepatitis C testing and treatment through the study, and specifically their views on starting hepatitis C treatment on the same day, prior to the RNA result being available, and ideas for improving models of care.

All participants of the hepatitis C trial will be informed that as part of the study, they may be invited to participate in qualitative interviews.

Interview participants will be purposively sampled to recruit participants that meet the following criteria:

- Participants in arm 1 who test RNA positive.
- Participants in arm 2 who test hepatitis C antibody positive at 5 min and RNA positive.
- Participants in arm 2 who test antibody positive at 5 min, RNA negative and did start treatment.
- Participants in arm 2 who test antibody positive at 5 min, RNA negative and did not start treatment (ie, those who received the medication but never took medication).

A total of approximately 50 participants will be recruited, with 20 participants in Armenia, 16 participants in Georgia and 14 participants in Tanzania. Where possible, interviews will be conducted by community researchers.

## Ethics and dissemination

### Funding

This study forms part of a Unitaid-funded portfolio of projects focused on preventing, testing and treating hepatitis C among people who inject drugs in LMICs (HCV Portfolio), which is being delivered by three Consortia. This study is being delivered by the Médecins du Monde Consortium, with collaborators from Burnet Institute, INPUD, University of Bristol and implementing partner organisations.

### Study management

The study is overseen by the Catalysing uptake of under‐utilised tools & treatment simplification for HepC (CUTTS HepC) project steering committee, convened by Médecins du Monde with at least one representative from each Consortium partner organisation. Study data management, study monitoring and independent monitoring are managed by Burnet. Relevant study investigators and data manager/statistician will have access to the final dataset. Access to the final dataset will otherwise be managed on request on a case-by-case basis.

### Ethical review

This study has undergone external scientific peer review and been approved by the Alfred Hospital Ethics Committee (Project #618/23, Project #340/24, Project #351/24), WHO Ethics Review Committee (ERC.0004066, ERC.0004123, ERC.0004276, ERC.0004257) and country-specific ethics committees (Armenia NCID Ethics Committee, Georgia Health Research Union, Tanzania NatHREC). Any amendments to the protocol will be communicated to relevant committees and investigators.

Informed consent will be obtained from all participants prior to enrolment. Potential participants will be presented with detailed information about the study procedures, objectives, risks and benefits; the consent form is provided in online supplemental material 4. Participants will be able to refuse or terminate participation at any time. Participants will be reimbursed US$4–US$10 at their baseline visit and US$4–US$10 for their SVR visit, for completion of the survey. Participants will be reimbursed US$30 for interview participation in Armenia and Georgia, and US$10 for interview participation in Tanzania. Reimbursement will be by cash in local currency. In Tanzania, participants attending Mukikute will also receive transportation support of US$1–US$4 for study visits (apart from when baseline and SVR questionnaire is completed during the study visit).

#### Data management

Online supplemental material 3 provides detailed description of data collected in each CRF and the participant survey questionnaire.

Data will be entered into and managed in Research Electronic Data Capture (REDCap), hosted and managed by Burnet Institute in Australia. REDCap is a secure web platform for building and managing online databases and surveys for research. In adherence to the European Union General Data Protection Regulation requirements, names and contact information will be securely stored in-country and will not be recorded in REDCap.

All CRFs and surveys are available in paper format in case of power or internet outage and will be stored securely at study sites.

Regular fortnightly data quality checks will be conducted throughout the study implementation period to ensure high-quality data. Quarterly site monitoring visits will also be conducted.

Quantitative data analysis will be performed in Stata SE V.17, the R Programming Language or equivalent analysis programme. Qualitative data analysis will be performed in NVivo, or other equivalent analysis programme and visualised using Lucidchart or equivalent programme.

### Adverse events and reporting

This study uses a drug with side effects that are already known.^49^ However, the study will trial a modified clinical pathway outside of current guidelines for HCV treatment in arm 2, that is, starting participants onto treatment drug without confirmation of HCV RNA status (current HCV infection) and without receipt of liver function and platelets investigation results to complete cirrhosis assessment.

It will therefore report adverse events that, in the opinion of the investigator, are not deemed to be expected or are increased in severity from prior observations. Adverse events considered related to the study drug administration will be captured up to 24 weeks after the last recorded study drug dose.

Burnet Institute, with Médecins du Monde as the study sponsor, is responsible for reporting adverse events and protocol modifications to Data Safety Monitoring Board and to the relevant Ethics Committees, where appropriate.

### Data monitoring

The trial will be monitored by an independent data safety and monitoring board with an independent statistician, clinical experts and community representatives, with at least one representative from each implementing country. There will be predefined periodic analysis if the data safety and monitoring board is concerned about any of the safety end points (serious adverse events, proportion ‘over-treated’, treatment failures).

Access to de-identified data will be limited to study investigators. The Data Safety Monitoring Board terms of reference were included in the ethics applications.

### Dissemination

The results from this study will be published and presented as peer-reviewed publications, lay summary reports (English and local languages), conference presentations, dissemination workshops with key stakeholders and with communities of people affected by HCV through relevant organisations/networks. Summary reports will be available on the Burnet Institute website, INPUD website, the Médecins du Monde website and at study sites. Results may also form part of PhD, Masters or Honours student theses. Authorship for publications arising from this study will adhere to Consortium authorship and publication policy, and the International Committee of Medical Journal Editors guidelines. The study protocol will be accessible via publication, and a full statistical analysis plan will be accessible as online appendix on the trial’s registration record.

All published quantitative and qualitative data will be non-identifiable aggregate data.

### Project timeline

Participant enrolment will close once the treatment initiation target has been met or by June 2026 (whichever is earlier). This allows sufficient time for enrolment and follow-up (minimum 6 months). The study will close in December 2026, or earlier if targets are met earlier.

The study opened recruitment in Armenia in October 2024, in June 2025 in Georgia and in August 2025 in Tanzania. Implementation is intended to continue up until December 2026.

## Discussion

The outcomes from both models of care are integral to supporting the national response to hepatitis C across the three implementing countries.

First, country-specific feasibility, acceptability, effectiveness and cost-effectiveness evidence is important to support scaled-up implementation and decentralisation of the simplified care model in Armenia and Tanzania specifically. Preparatory work for the study has further simplified the care model in Georgia for all harm reduction sites by removing the requirement for on-treatment monitoring.

Second, the utility of the 5 min read-time component to support same-day treatment initiation will be dependent on the impact on linkage to treatment, the proportion overtreated and the relative cost-effectiveness of this strategy. The potential utility will also differ across countries based on background prevalence and incidence of HCV, and treatment rates, and the relative cost of HCV RNA tests compared with HCV treatment.

While we are evaluating the feasibility and scale-up requirements of these models of care to inform future scale-up, the implementation as part of a research study with relatively few participants compared with national programmes limits its direct replicability for scaled-up implementation.

The outcomes of this study will support the ongoing decentralisation and scaled-up implementation of national hepatitis C response in the three implementing countries and provide evidence to support efforts in other LMICs.

## Supplementary material

10.1136/bmjopen-2025-114119 Supplementary Material 1

10.1136/bmjopen-2025-114119 Supplementary Material 2

10.1136/bmjopen-2025-114119 Supplementary Material 3

10.1136/bmjopen-2025-114119 Supplementary Material 4

10.1136/bmjopen-2025-114119 Supplementary Material 5
