# Supplementary material for: Non-randomised trial of a hepatitis C same-day test and treat model using antibody test only for people who inject drugs in Armenia, Georgia and Tanzania: a CUTTS HepC study protocol
Source: BMJ Open. 2026 Mar 24;16(3):e114119. doi: 10.1136/bmjopen-2025-114119 (PMC13034256; doi:10.1136/bmjopen-2025-114119)
Supplement: Supplementary Material 1 [file bmjopen-16-3-s001.docx]

**Supplementary Material 1 – Summary of HIV RDTs, HBsAg RDTs, HCV RNA tests and DAAs**

|  | **HIV RDT** | **HBsAg RDT** | **HCV RNA test** | **DAAs** |
| --- | --- | --- | --- | --- |
| Armenia | Brand: InTec INC, Abbott , Standard Q™  Test Name: One step anti-HIV 1&2 test, Rapid diagnostic test HIV 1/2, HIV/Syphilis combo test | Brand: InTec INC,  Factor-Med Production production LLC, Russia , Accu-Tell  Test Name:  One step HBsAg test,  HBsAg Test**,** HBsAg Cassette | Cepheid GeneXpert HCV VL | SOF/VEL  Manufacturer:  Velpanat, Natco Pharma Ltd  MyHep ALL, Mylan Pharmaceuticals Private Limited |
| Georgia | Brand: InTec  Test Name: ONE STEP Anti-HIV (1&2) Test HIV | Brand: CTK Biotech, Inc, USA  Test Name: On Site Rapid Test | Cepheid GeneXpert HCV VL  HCV Real-TM Quant Dx CE-IVD, Sacace Biotechnologies | SOF/VEL  Manufacturer:  Mylan MyHep All™ |
| Tanzania | Brand: ABBOTT  Test Name: HIV BIOLINE | Brand: MERISCREEN  Test Name: Hepatitis B surface antigen | Cepheid GeneXpert HCV VL | SOF/VEL  Manufacturer:  Mylan Laboratories Limited, India  &  Epclusa, Patheon Inc, Canada |
