# Supplementary material for: Non-randomised trial of a hepatitis C same-day test and treat model using antibody test only for people who inject drugs in Armenia, Georgia and Tanzania: a CUTTS HepC study protocol
Source: BMJ Open. 2026 Mar 24;16(3):e114119. doi: 10.1136/bmjopen-2025-114119 (PMC13034256; doi:10.1136/bmjopen-2025-114119)
Supplement: Supplementary Material 2 [file bmjopen-16-3-s002.pdf]

Supplementary Material 2 – Feasibility and Implementation Requirements Overview

**Components**	**Outcomes (Barriers / Enablers)**	**Data Sources**	**Consolidated Framework for Implementation Research (CFIR) DOMAIN and constructs**
	Barriers and enablers specific to POC testing process steps		
Point-of-care testing (Rapid HCV Antibody Test)	Collecting the sample	Key informant interviews with study clinicians<br><br>Site monitoring checklists (incl. observations)	INNOVATION: Complexity; INDIVIDUALS, CHARACTERISTICS: Capability
	Operating the device and interpreting result	Key informant interviews with study clinicians<br><br>Site monitoring checklists (incl. observations)	INNOVATION: Complexity; INDIVIDUALS, CHARACTERISTICS: Capability
	Participating in quality assurance	Key informant interviews with study clinicians<br><br>Site monitoring checklists (incl. QA program results)	INNOVATION: Complexity; INDIVIDUALS, CHARACTERISTICS: Capability INDIVIDUALS, ROLES: Other Implementation Support
	Disclosing results to participant	Key informant interviews with study clinicians	INNOVATION: Complexity;
	Understanding results & implications	Qualitative interviews with trial participants	INNOVATION: Complexity;<br><br>CHARACTERISTICS: Need, Capability, Opportunity, Motivation
Treatment work-up and initiation	Performing decompensated	Key informant interviews with	INDIVIDUALS, CHARACTERISTICS

	cirrhosis exclusion assessment	study clinicians	: Capability<br>INNER SETTING: Access to Knowledge & Information
	Conducting rapid HIV, HBV and pregnancy tests	Key informant interviews with study clinicians<br><br>Site monitoring checklists (incl. observations)	INNER SETTING DOMAIN; Structural Characteristics, Physical Infrastructure, IT Infrastructure, Available Resources – funding, space.
	Prescribing treatment	Key informant interviews with study clinicians	INNOVATION: Complexity;
		Qualitative interviews with trial participants	INNOVATION: Complexity;<br><br>CHARACTERISTICS : Need, Capability, Opportunity, Motivation
	Referral to specialist for review (uncontrolled/ new HIV infection, HBsAg positive, TB infection, decompensated cirrhosis)	Key informant interviews with study clinicians<br><br>Reflection and learnings workshops	OUTER SETTING: Partnerships & Connection
Treatment monitoring and dispensing	Contacting participants after starting treatment, if RNA negative	Case report forms<br><br>Key informant interviews with study clinicians<br><br>Reflection and learnings workshops	INNER SETTING: Structural Characteristics, Work Infrastructure<br>INNER SETTING: Cultural, Recipient-Centredness
	Stopping treatment once notified they were RNA negative – understanding implications	Qualitative interviews with trial participants	INNOVATION: Complexity;<br><br>CHARACTERISTICS : Need, Capability, Opportunity,

			Motivation<br><br>INNER SETTING: Access to Knowledge & Information
	Dispensation of medication	Key informant interviews with study clinicians<br><br>Reflection and learnings workshops	INNER SETTING: Structural Characteristics, Work Infrastructure<br>INNER SETTING: Available Resources
	Treatment monitoring	Qualitative interviews with trial participants<br><br>Key informant interviews with study clinicians<br><br>Reflection and learnings workshops	INNOVATION: Complexity;<br><br>CHARACTERISTICS: Need, Capability, Opportunity, Motivation<br><br>IMPLEMENTATION PROCESS: Tailoring Strategies, Reflecting & Evaluating
Cross-cutting barriers and enablers to implementation of models of care	Competing clinical and operational demands	Process Activity & Monitoring Log<br><br>Key informant interviews with study clinicians<br><br>Reflection and learnings workshops	INNER SETTING: Relative Priority;
	Difficulty integrating hepatitis C models of care into workflow	Process Activity & Monitoring Log<br><br>Key informant interviews with study clinicians<br><br>Reflection and learnings workshops	INNOVATION: Complexity;<br><br>IMPLEMENTATION PROCESS: Assessing Context, Reflecting & Evaluating
	Difficulty obtaining appropriate	Process Activity & Monitoring Log	OUTER SETTING: Policies & Laws

permissions	Key informant interviews with study clinicians<br><br>Reflection and learnings workshops	
Study clinicians trust and knowledge of the models of care and same-day treatment	Key informant interviews with study clinicians<br><br>Reflection and learnings workshops	INNOVATION: Innovation Source, Innovation Evidence-base, Innovation Relative Advantage<br>INNER SETTING: Access to Knowledge & Information, Culture
Difficulty adapting clinic set-up to accommodate models of care	Process Activity & Monitoring Log<br><br>Site monitoring checklists	INNER SETTING: Structural Characteristics<br>INNER SETTING: Available Resources
