# Supplementary material for: Non-randomised trial of a hepatitis C same-day test and treat model using antibody test only for people who inject drugs in Armenia, Georgia and Tanzania: a CUTTS HepC study protocol
Source: BMJ Open. 2026 Mar 24;16(3):e114119. doi: 10.1136/bmjopen-2025-114119 (PMC13034256; doi:10.1136/bmjopen-2025-114119)
Supplement: Supplementary Material 3 [file bmjopen-16-3-s003.pdf]

Supplementary Material 3 – Case Report Forms and Survey Data Collection Overview

**Summary of CRFs & items**			
CRF #	Study Timepoint (Contact #)	CRF	CRF items
0	0	Pre-enrolment eligibility screening checklist	• Aged 18 years or older;<br>• Attending site for needle / syringe program OR self-reports ever injecting drugs;<br>• Not currently on or previously had treatment for hepatitis C; and<br>• Able and willing to provide informed consent in local language.<br>• Citizen of country (Armenia & Georgia)
1	1	Registration	• Participant demographics: age, sex/gender (CRF, entered into REDCap); and<br>• Participant contact details and secondary contact details (stored locally at study site, password protected excel sheet).
2	1	Baseline	• OraQuick Test Results: result read at 5 minutes, result read at 20 minutes<br>• Other rapid test results: HIV, HbsAg<br>• Pregnancy test (urine dip-stick test)<br>• Medical history via participant self-report to exclude previous or current treatment for hepatitis C, and through review of any medical records kept by the service<br>• Opioid agonist therapy: ever, currently, what type (e.g. methadone, buprenorphine, long-acting buprenorphine);<br>• Injecting drug use behaviours: frequency of injecting, most commonly used drug; and<br>• Alcohol use: AUDIT-C.<br><br>**Clinical Exclusion Criteria:**<br>• History of decompensated cirrhosis of the liver<br>• Women who are or may be pregnant (self-report and pregnancy test) or breast-feeding (self-report)<br>• Other significant co-morbidities such as uncontrolled HIV infection, history of

			renal dysfunction, tuberculosis infection, or chronic hepatitis B infection<br>• Unable / unwilling to stop any contraindicated medications / supplements<br>•
3	1 (arm 2) / 2 (arm 1)	Treatment Plan	• Hepatitis C RNA test result (Arm 1 only)<br>• Details on return of hepatitis C RNA result to participant (Arm 1 only)<br>• Medical history via participant self-report and through review of any medical records kept by the service<br>• Other laboratory investigations (Arm 1 only)<br>• Cirrhosis assessment outcome<br>• Any referrals to specialists<br>• Treatment plan<br>• (Study exit details if applicable, recorded in CRF9)
3.a	2	(Arm 2 Only) Revisions to Treatment Plan	• Hepatitis C RNA test result<br>• Details on return of hepatitis C RNA result to participant<br>• Other laboratory investigations<br>• Cirrhosis assessment outcome<br>• Any referrals to specialists<br>• Treatment plan & adjustments<br>• Treatment adherence
4	3, 4, 5	Monitoring / Dispensing	• Adherence, side effects<br>• Any adverse events<br>• Issues with lost medication<br>• Any revisions to treatment plan (e.g. if participant ceases treatment or needs to re-start treatment after missed doses)<br>• Next scheduled contact (remote contact or in-person); request for peer worker support
5	6, 7	SVR assessment	• Date participant attended for SVR assessment phlebotomy<br>• SVR test result and outcome<br>• Any counselling / referrals for ongoing care

			• Harm reduction counselling; explanation re: re-treatment if necessary
6	Any timepoint	Peer worker interactions	• Date<br>• Purpose of contact (treatment dispensing, reminders about appointments, monitoring check-in)<br>• Mode of contact (in-person, virtual)<br>• Notes about contact and any actions
7	Any timepoint	Participant contact attempts	• Date<br>• Purpose of contact<br>• Mode of contact<br>• Outcome of contact
8	Any timepoint	Specialist referral form	• Investigations<br>• Revised Treatment Plan<br>• Any other advice<br>• Study exit details (if applicable)
9	Any timepoint	Study exit form	• Reason for exit: withdrawn due to medical issue / ineligible, withdrawn due to LTFU, withdrawn due to participant choice to stop participation<br>• Details<br>• Timepoint<br>• Whether participant withdrew consent for study data to be utilised
10	Any timepoint	Adverse event reporting form	• Date reported<br>• AE onset<br>• AE stop<br>• Location AE occurred<br>• Unexpected AE<br>• Description of AE<br>• Category of AE<br>• Relationship of event to intervention<br>• Intervention discontinued due to AE<br>• Actions taken to treat AE<br>• Relevant tests and other participant history

**Survey #**	**Timepoint**	**Domains**
1	Baseline (Contact 1 and/or 2)	• Hepatitis C medical and testing history;

		• Risk factors for hepatitis C; including injecting drug use behaviours<br>• Out of pocket expenses incurred to attend contact 1;<br>• Work productivity and employment: frequency of work, current employment status;<br>• Health related quality of life: EQ-5D-5L<br>• Other demographics: incarceration history, housing;
2	SVR (Contact 6 and/or 7)	• Other demographics: incarceration history, accommodation;<br>• Substance use (alcohol, other drug use)<br>• Work productivity and employment: frequency of work, current employment status;<br>• Health related quality of life: EQ-5D-5L
