# Supplementary material for: Non-randomised trial of a hepatitis C same-day test and treat model using antibody test only for people who inject drugs in Armenia, Georgia and Tanzania: a CUTTS HepC study protocol
Source: BMJ Open. 2026 Mar 24;16(3):e114119. doi: 10.1136/bmjopen-2025-114119 (PMC13034256; doi:10.1136/bmjopen-2025-114119)
Supplement: Supplementary Material 4 [file bmjopen-16-3-s004.docx]

**Informed Consent form for Participation in CUTTS HepC: Simplifying Hepatitis C Pathways Trial (Arm 1 & 2)**

**Name of Principal Investigator:** Prof. Margaret Hellard

**Name of Organization:** Burnet Institute, Australia

**Name of Sponsor:** Médecins du Monde

**Name of Proposal and version:** CUTTS HepC - Simplifying hepatitis C pathways for people who inject drugs in Armenia, Georgia, and Tanzania, V1.3, 11 April 2024

The [country] Ministry of Health has provided permission for this study to be delivered.

**This Informed Consent Form has two parts:**

- **Information Sheet (to share information about the research with you)**
- **Certificate of Consent (for signatures if you agree to take part)**

**You will be given a copy of the full Informed Consent Form**

**PART I: Information Sheet**

1. **Purpose of this research**

Hepatitis C is a virus that can lead to liver damage. There is medication that can cure hepatitis C in most people which has very few side effects, and stop any further liver damage. Before starting this medication, you usually need two tests to find out if you have hepatitis C. The first test finds out if you have *ever* had hepatitis C (which means you may or may not still have it) and is called an antibody test. The second test sees if you currently have hepatitis C and is called a ribonucleic acid test (RNA test). The issue with this is that the RNA test is expensive, it can take a few days to get your results back and is not available in all places.

The aim of this project is to explore the way we could test for hepatitis C and start people on hepatitis C treatmen to make it easier and cheaper for you. As part of this, we will look at if this different way of testing and treatment can increase the number of people who start treatment, reducing the number that do not access treatment after getting tested.

The antibody test is usually finished in 20 minutes, but there have been some studies that show that if the result is read at 5 minutes, it can tell us if you have current hepatitis C infection. This means the expensive RNA test will not be needed and you can start treatment earlier. This study will investigate whether offering hepatitis C treatment on the same day will increase the number of people who are diagnosed, treated and cured for hepatitis C.

1. **Your voluntary participation**

We are asking all adults who come to [service] for needle/syringe program or who self-report injecting drugs to participate in this research. We are inviting people who inject drugs because they have a higher chance of having hepatitis C.

Your participation in this research is entirely voluntary and it is your choice whether to participate or not. Regardless of if you choose to participate or not, you will still be able to use all of the services at [service/clinic] which you usually have access to.

You should know that if you choose not to participate in this research project, you may not be able to access hepatitis C treatment at this service as it may not be available outside of this research yet [insert site specific information here]. If you choose not to participate, we will tell you the options for getting treatment from somewhere else if it is available.

You may change your mind later and stop participating even if you agreed earlier to participate. This will not change your access to the usual care at this service, but it might change your access to hepatitis C testing and treatment.

1. **What you will do in this research**

Groups

If you choose to participate, you will be put into one of two groups, you will not get to choose which group you are put into. The groups are:

1. Arm 1: you will be tested for hepatitis C by having both the antibody and RNA tests done before treatment begins
2. Arm 2: you will have your antibody test read earlier than usual at 5 minutes and will start treatment based on this result on the same day. If in this group, you will also have the RNA test done to confirm that the first test was correct and receive this result after taking home the treatment medication.

The type of antibody test we will do in this study is called an OraQuick® Rapid hepatitis C Test. For the rest of this document, we will call it an OraQuick test.

More detail on what you will do in each group is listed below.

Appointments

The amount of times you will need to come to [clinic] depends on which Arm you are in.

- Arm 1: you will need to come to [clinic] in person a minimum of 4 times in a 9-month period
- Arm 2: you will need to come to [clinic] in person a minimum of 3 times in a 9-month period

Each visit will take up to 1 hour. Any appointments completed over the phone will take approximately 15-30 minutes.

Treatment

The drug we are using in this research is called SOF/VEL (sofosbuvir + velpatasvir), it is a direct-acting antiviral used to treat hepatitis C infection. The drug has been proven to be safe and effective, and is approved by authorities for use globally, including recommended in WHO guidelines.

The drug is approved in [COUNTRY]. You should know that the drug (SOF/VEL) has the following known side effects: headache, fatigue, low blood iron (anaemia), nausea, insomnia, diarrhoea, weakness, rash and depression.

In this study, you will take this medication orally once a day. You will take this medication at home and will not need a nurse or doctor to be present.

Appointment activities and tests

Appointment 1:

- You will have to come to [clinic] and have a small amount of blood taken from your finger and a small amount of blood taken from your arm with a syringe (approximately 25mL in total). This blood will be used to test for
  - Hepatitis C through the OraQuick test and the RNA test
  - Hepatitis B
  - Other liver function tests
- A doctor will do a physical examination to check your liver health
- You will be asked a few questions about your general health, your injecting drug use behaviours and your hepatitis C history
- If you are in Arm 1, your OraQuick test will be read at 20 minutes. If it is positive, your blood sample will be sent for RNA testing. You will receive the results within 2 weeks of your first appointment.
- If you are in Arm 2, your OraQuick test will be read at 5 minutes. If it is positive, you will start treatment for hepatitis C that same day. If the OraQuick test turns positive at 5 minutes, or later at 20 minutes, your RNA test will also be done and you will receive the results within 2 weeks of your first appointment.

Appointment 2:

- If you are in Arm 1, you will come back to [clinic] and receive your test results. If they are positive, you will begin treatment.
- If you are in Arm 2, you will receive a phone call or be contacted by service outreach staff and told to come back to the clinic if needed, or to stop / continue treatment from that day. You will also receive your other test results.

Appointment 3:

- After 4 weeks of treatment, you will return to [clinic] to pick up 8 more weeks of treatment
- You will be asked some questions about how treatment is going for you

Visit 4:

- After 8 weeks of treatment you will receive a phone call or be asked to come to clinic to answer some questions about how treatment is going for you
- The same thing will happen at week 12 of treatment

Visit 5:

- 4 – 20 weeks after you finish treatment, you must come back to [clinic] to have another blood test that checks if you currently have hepatitis C infection to check if the treatment worked. Approximately 10mL of blood will be taken for this test.
- At the visit to take the blood sample, we will ask you some questions in a survey about your quality of life, employment and your current living situation, and your injecting drug use behaviors

Any leftover blood samples will be destroyed after tests are successfully done by clinic or laboratory.

Here is a diagram that shows the two different groups and what will happen:


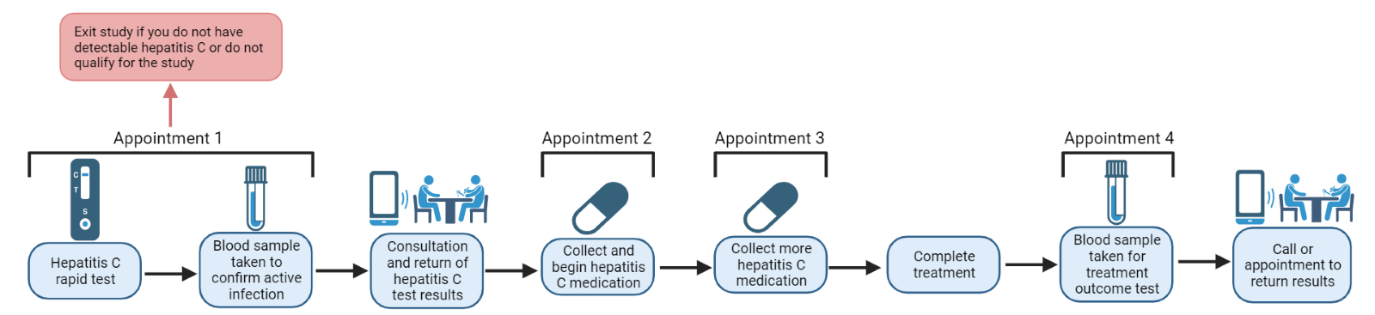


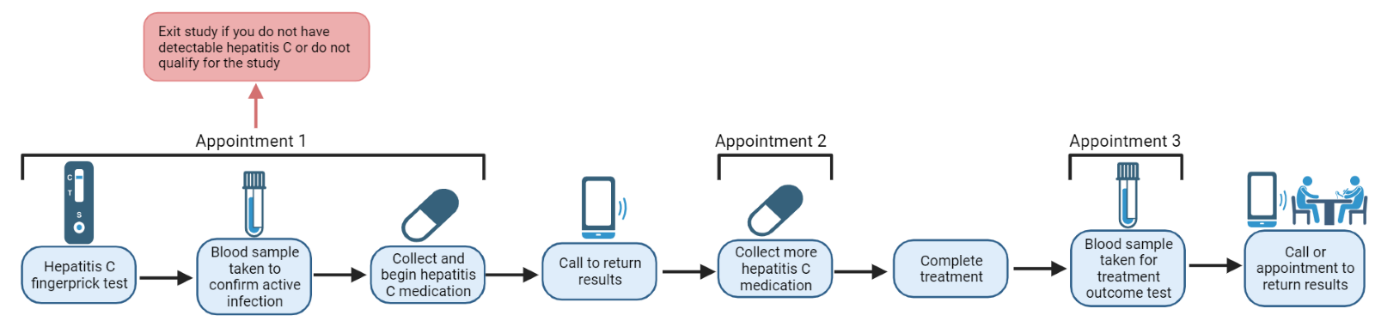

As part of this study, research staff will observe some appointments to see how long the tasks completed in the appointment usually take. This will help us understand whether this new way of testing is cost-effective. Only a small number of participants will have their appointment observed by a research staff member. If your appointment is selected for observation, the research staff will first ask you if you are comfortable with this. You have the right to decline without any negative consequences.

If a staff member observes your appointment, they will only be recording the tasks completed from a checklist, the time taken and any comments provided by clinicians on whether this amount of time was the ‘average’ for their consultations most days. Your appointment will remain confidential and none of your personal details will be recorded.

Interviews

Some people will be invited to do an interview with a researcher on their experience with starting treatment. If you agree to be contacted about an interview, you will be taken through this similar process of informed consent to find about the study before you agree to do the interview.

**Pregnancy and breastfeeding**

To participate in this study, you must not be currently pregnant or breastfeeding. This is to protect your safety and the safety of your child as the medication we are using in this study is not recommended for pregnant or breastfeeding women by the World Health Organisation. This is because there is not yet enough data to show if it is safe or not Female participants (aged 18 - 49 years) enrolled in the study will also have a pregnancy test completed by a study nurse or doctor before beginning treatment. While participating in the study, contraception must be used to avoid pregnancy.

If you do become pregnant while on this medication, you will have an appointment with a specialist and your specialist and the study doctor will also discuss the situation, and together you will decide whether you want to continue on treatment or not. Although it is not recommended to start treatment while pregnant, there is some evidence that shows that it is not dangerous to continue treatment .

If you choose to stop treatment, you will be asked to return later to have a RNA test to see if you still have hepatitis C. You will also be contacted by study staff and asked some questions about your health during and after your pregnancy.

If you choose to continue on treatment, you will follow the same process as the other individuals in the study. Study staff will contact you during and after your pregnancy to see if you are experiencing any side effects of medication.

The information collected about your health during and after your pregnancy will be submitted to an online database to help gather evidence about whether taking treatment is safe while pregnant. None of your personal information will be sent to this database and it will be completely anonymous.

If you start breastfeeding after starting treatment, a discussion will be organised with a clinician and you will decide together whether to continue breastfeeding while on treatment or not.

**Side Effects**

As already mentioned, this drug can have some unwanted effects. It can cause headache, fatigue, low blood iron (anaemia), nausea, insomnia, diarrhoea, weakness, rash and depression. It is possible that it may also cause some problems that we are not aware of. However, we will follow you closely and keep track of any unwanted effects or any problems. If necessary, we will discuss it together with you and you will always be consulted before we move to the next step.

SOF/VEL is a very safe medication and there are no expected impacts of taking this medication if your hepatitis C RNA test result is negative (e.g., you do not have current infection).

**Risks of participating**

By participating in this research, it is possible that you will be at greater risk than you would otherwise be. There is a risk that you will start treatment and it will make any existing liver disease worse. The possibility of this happening is very low. We will try decrease the chances of this event occurring by asking you questions about your medical history and by the study doctor performing a physical examination to check for liver disease, and by doing some blood tests that look at your liver function. If something unexpected happens, we will provide you with / advise you on where to access urgent medical care and appropriate referrals for ongoing care.

There is a chance that you will start treatment when you do not have current hepatitis C infection. The risks to you if you have treatment without infection are the same as if you did have the infection except that we would not know your liver function test results before you start treatment; as above, you would be at risk of making any existing liver disease worse. If you do not have infection, you are less likely to have liver disease (you may still have it from alcohol use or other infections). The difference between Arm 1 and Arm 2 (same-day treatment) is that we will not know your liver function test results before giving you treatment.

Apart from this, the possible risks of participating in this research include:

- Discomfort when answering questions on illicit drug use
- Discomfort at having blood samples taken for hepatitis C and HIV tests
- Breach of privacy / confidentiality of information provided on illicit drug use
- Breach of privacy / confidentiality of any additional contact information provided to service providers (if not already collected by service)

If you experience any discomfort answering questions on illicit drug use and would prefer not to provide a response, you can choose to skip these questions in the survey. You will be reminded of this option when you are asked these sets of questions. Trained staff will be available to provide extra support after being asked or answering these questions if you need, and to provide you with the details of other service available to you.

The staff taking blood samples for hepatitis C and HIV tests will be appropriately trained to reduce the risk of discomfort. If you experience any discomfort or harms at the time of taking blood, please tell the staff.

We will follow strict procedures to keep your information safe and secure to reduce the risk of a breach of privacy / confidentiality. The contact details provided to the study staff will be kept separately from your survey answers and test results. Only specific study staff will be able to access contact information.

In some rare cases, we may have to share confidential information if we:

- think you may seriously harm yourself or someone else;
- have been asked to provide this information by a court of law; or
- learn information concerning the protective safety of children.

**Benefits** **of participating**

If you participate in this research, you will be able to access hepatitis C testing and treatment (if eligible) for free. There may not be any other direct benefits for you, but your participation will help us understand how to increase access to testing and treatment here and in other countries too.

**Reimbursements**

You will not be given any other money or gifts to take part in this research.

**Your information**

With this research, something out of the ordinary is being done at this service by providing hepatitis C treatment. It is possible that if others in the community or at this service are aware that you are participating, they may ask you questions. We will not be sharing the identity of those participating in the research.

Study clinicians involved in providing you hepatitis C testing and treatment during this study may also look at the records already kept by this service about your medical history to collect information to complete the study forms and to get a complete picture of your medical history. The information they look at and include in the study forms may include your previous hepatitis C testing and treatment history, any details on your HIV treatment and viral load suppression, and any comments on the stage of any diagnosed liver disease, among other items. Same as when you visit a doctor usually, the study staff (doctor/nurse only) may contact your treating physicians for further information about your medical history and to update them on your current care, or refer you to a specialist for a review.

The information that we collect from this research project will be kept confidential. Information about you that will be collected during the research will be put away and no-one but the researchers or staff at this service involved in delivering the research will be able to see it. Any information about you will have a number on it instead of your name. Only the researchers will know what your number is and we will keep that information up safe by storing it in cabinets with locks or on a password protected computer file. It will not be shared with or given to anyone except the research team, the Data Safety Monitoring Board, and your other clinicians (with your permission).

**What will happen to the study results**

The knowledge that we get from doing this research will be shared in many formats:

- Presentations at scientific meetings
- Publications in health journals
- Workshops and meetings with organisations and governments

Results may also form part of further education research projects.

We will use these results to encourage people to support hepatitis C care and to increase access to hepatitis C medication. We will make sure that all study results respect the views and interests of local communities and all of the people who participate. The research team will make sure that none of the work reinforces negative stereotypes or attitudes towards people who inject drugs.

**You can see the results**

The results will be made available at your study site [this service] towards the end of the study or once it has finished. The study will be completed over a two-year period. If you wish to access the results, you may visit [this service] once available and request access from the site staff. This summary will also be freely available on the Medecins du Monde website, and the staff at this service can help you find a weblink to the summary report.

This study is intended to benefit the communities being researched. Results from this research project will be used to advocate for further funding to support sustainable service provision. Great attention will be given to ensuring that all findings are communicated in ways which respect the authentic views and interests of the local communities participating in the study.

**You do not have to take part in this research**

You do not have to take part in this research if you do not wish to do so. You may also stop participating in the research at any time you choose by telling the study staff. You may not be able to access hepatitis C testing or treatment at no cost at this site. It is your choice and all of your rights will still be respected.

If you later decide to stop participating in the study, you will be given the option to withdraw your consent and your study data. This will be possible, unless we have already started analysing the data (which will start two years after the study begins); in that case, we will no longer be able to remove your data. We are unable to remove your data after analysis has begun because when analysing your data, we will remove any information that could potentially identify you. This means that we will not be able to trace the data back to you to remove it.

**Alternatives to Participating**

If you do not wish to take part in the research, you will be provided with information about how to access hepatitis C testing and treatment elsewhere, if available. This will likely be at a hospital in the capital city. You would receive the same or similar treatment drug recommended by global guidelines.

**Who to Contact**

If you have any questions you may ask them now or later, even after the study has started. If you wish to ask questions later, you may contact any of the following:

[Name of local investigator]

[Organisation]

[Telephone number]

[e-mail address]

Principal Investigator: Prof. Margaret Hellard

Organisation: Burnet Institute

e-mail address: [margaret.hellard@burnet.edu.au](mailto:margaret.hellard@burnet.edu.au)

Study Co-ordinator: Dr. Bridget Draper

Organisation: Burnet Institute

e-mail address: bridget.draper@burnet.edu.au

**This proposal has been reviewed and approved by [name of the local IRB], which is a committee whose task it is to make sure that research participants are protected from harm. If you wish to find about more about the IRB, contact [name, address, telephone number.]). It has also been reviewed by the Ethics Review Committee of the World Health Organization (WHO), which is funding/sponsoring/supporting the study.**

You can ask me any more questions about any part of the research study, if you wish to. Do you have any questions?

**PART II: Certificate of Consent**

I have been invited to participate in a study where I will be offered hepatitis C testing and treatment to compare two different pathways to getting onto treatment, and to look at the accuracy of a new test. I know that I may be put in the group where I will be offered same-day treatment, before I know the results from tests on whether I have current infection and on my liver function. I will have blood samples taken for testing at least twice, and up to six times. I will be told what the tests are for each time. I will be offered hepatitis C treatment, where I will be asked to take one or two tablets daily for 12 weeks and then return for a test to find out if treatment worked for me. The study doctor and the extra surveys will ask me questions about my medical history, injecting drug use, my current living situation and my quality of life, but I know that I do not have to answer all these questions if I do not feel comfortable.

**I have read the foregoing information, or it has been read to me. I have had the opportunity to ask questions about it and any questions that I have asked have been answered to my satisfaction. I consent voluntarily to participate as a participant in this research.**

**Print Name of Participant__________________**

**Signature of Participant ___________________**

**Date ___________________________**

**Day/month/year**

**If illiterate**

A literate witness must sign (if possible, this person should be selected by the participant and should have no connection to the research team). Participants who are illiterate should include their thumb-print as well.

**I have witnessed the accurate reading of the consent form to the potential participant, and the individual has had the opportunity to ask questions. I confirm that the individual has given consent freely.**

**Print name of witness_____________________ AND Thumb print of participant**

**Signature of witness ______________________**

**Date ________________________**

**Day/month/year**

**Statement by the researcher/person taking consent**

I have accurately read out the information sheet to the potential participant, and to the best of my ability made sure that the participant understands that the following will be done:

1. hepatitis C and other pre-treatment tests

2. surveys administered by staff and collection of clinical information by study clinicians

3. offer of hepatitis C treatment, including before knowing if participant has current infection

4. one re-tests for hepatitis C to check if treatment worked

I confirm that the participant was given an opportunity to ask questions about the study, and all the questions asked by the participant have been answered correctly and to the best of my ability. I confirm that the individual has not been coerced into giving consent, and the consent has been given freely and voluntarily.

**A copy of this ICF has been provided to the participant.**

**Print Name of Researcher****/person taking the consent________________________**

**Signature of Researcher /person taking the consent__________________________**

**Date ___________________________**

**Day/month/year**
