# Supplementary material for: Non-randomised trial of a hepatitis C same-day test and treat model using antibody test only for people who inject drugs in Armenia, Georgia and Tanzania: a CUTTS HepC study protocol
Source: BMJ Open. 2026 Mar 24;16(3):e114119. doi: 10.1136/bmjopen-2025-114119 (PMC13034256; doi:10.1136/bmjopen-2025-114119)
Supplement: Supplementary file 6 [file bmjopen-2025-114119.reviewer_comments.pdf]

PEER REVIEW HISTORY

BMJ Open publishes all reviews undertaken for accepted manuscripts. Reviewers are asked to complete a checklist review form and are provided with free text boxes to elaborate on their assessment. These free text comments are reproduced below.

ARTICLE DETAILS

Title (Provisional)

A non-randomised trial of a hepatitis C same-day test and treat model using antibody test only for people who inject drugs in Armenia, Georgia, and Tanzania: a CUTTS HepC study protocol

Authors

Draper, Bridget Louise; Flynn, Mia; Schroeder, Sophia; Wisse, Ernst; Aikaeli, Faith; Han, Zin Mar; Ayako, Maureen; Bajis, Sahar; Butsashvili, Maia; Davtyan, Kamo; Kordzadze, Tea; Lamand, Pauline; Luhmann, Niklas; Sargsyan, Knarik; Senkoro, Mbazi; Scott, Nick; Stone, Jack; Vickerman, Peter; Voloshin, Anita; Walker, Josephine; Madden, Annie; Stoové, Mark A; Hellard, Margaret; Pedrana, Alisa; Hepatitis C Models of Care Study Team, CUTTS HepC Consortium

---

VERSION 1 - REVIEW

**Reviewer**	**1**
**Name**	**Torgersen, Jessie**
**Affiliation**	**University of Pennsylvania, Medicine**
**Date**	**30-Dec-2025**
**COI**	**None**

The manuscript by Draper, et al., presents an exciting protocol of a non-randomized trial of a hepatitis C virus (HCV) test-and-treat strategy using same-day point of care testing (POCT) for HCV antibody within centers providing care for people who inject drugs (PWID) across three different low- and middle-income countries (LMIC). The authors are leveraging previously published work by Smookler, et al. noting people with HCV viremia were identified by an early-read time of the POCT OraQuick HCV antibody assay. While the authors appropriately note the sensitivity of the 5-minute reading time is 100%, the positive predictive value (PPV) was only 62% and may result in overtreatment when using this threshold to initiate HCV treatment. The protocol includes a robust analytic plan to evaluate the feasibility, effectiveness, as well as cost-benefit analysis to inform scalability of this program to other regions. The authors provide a clear justification and rationale for the inclusion/exclusion criteria, study site selection, workflow, and data collection. I provide the following suggestions to improve the clarity of the methods detailed:

Major Suggestions:

- Page 13 line 31 suggests many LMIC provide HCV antibody tests are offered free-of-charge as part of harm reduction. Are these antibody tests the POCT diagnostics included in the protocol, such as the OraQuick HCV antibody assay? If not, what is the anticipated cost of the OraQuick assay and how are these being provided for the study activities?

- The protocol is very clear regarding workflow for confirmatory testing and treatment dispensation. How will treatment interruptions be addressed in the protocol? Is there a certain time point of missed treatment after which the participant will not be provided additional fills of sofosbuvir/velpatasvir? Similarly, how will participants with errors in confirmatory testing be managed (i.e., if confirmatory HCV RNA canceled/not completed after initiation of sofosbuvir/velpatasvir)?

- Table 2 reports different lower end thresholds for FIB-4 to exclude cirrhosis, specifically 1.25 and 1.45, while the text references 1.45 only for the lower threshold. Please revise Table 2 if the 1.25 value is an error. If not an error, please provide additional rationale for using the 1.25 threshold.

- Data will be entered into REDCap; however, it was not clear if data be managed centrally or at each individual site. Consider including a statement about use of data management center, if applicable.

Minor Suggestions regarding Additional Analyses:

I can appreciate that there will be many additional analyses planned, both a priori and post hoc) that, for the sake of word count, cannot be included in the protocol. There are three aspects that I believe may warrant explicit comment and encourage the authors to consider the following:

- Will QraQuick performance characteristics be evaluated by HIV status? The validation work by Smookler included only 59 people with HIV across the clinic and Madrid cohorts, the majority of whom had CD4 cell count >200 (where these data were reported). Considering the prevalence of HIV is higher in the regions included in this study, the activities proposed may allow for further evaluation of the performance characteristics by HIV status.

- Will there be an analysis of treatment completion as the 12-week treatment course prescribed? Page 34, line 32 describes secondary outcomes, but only defines completion of treatment as completing >=8 weeks of sofosbuvir/velpatasvir. As the prescribed sofosbuvir/velpatasvir course of 12 week, consider indicating if competing the entire prescribed course will be part of the analysis.

- Will there be quantitative/qualitative assessment of uptake of services such as HIV PrEP and/or HBV vaccination? Co-location of care for PWID is key to reduce barriers to and understanding the acceptability and uptake of these related services to prevent other blood-borne viral infections can inform implementation efforts.

Suggestions for Text Clarification/Edits:

- Throughout the text, there is a shift between MAT and OAT; however, only OAT is defined in the abbreviations (page 18, line 36). Consider revising to keep the abbreviations/terminology consistent.

- The authors focus on the sensitivity and specificity of the OraQuick assay (page 29, line 24); however, the PPV will be a key measurement, particularly as it will vary by prevalence of HCV viremia. This is a strength of the analysis - consider revising the text to highlight all performance characteristics of OraQuick will be evaluated, not just sensitivity and specificity.

- Introduction page 12, line 14 (4th sentence): “Globally, people who inject drugs are a key population groups affected by hepatitis C…” – remove the word “groups” as it redundant.

- Introduction page 12, line 16 (5th sentence): “Transmission risk in this groups predominantly due to the re-use of injecting equipment is re-used.” Consider revising to, “Transmission risk in this group is predominantly due to the re-use of injecting equipment.”

- Page 18, line 35: capitalize the name of the hospital Muhimbili National Hospital in the parentheses.

-Page 23, line 35: “those who return a non-reactive HBsAg RDT and have no evidence of prior hepatitis B vaccination will be offered vaccination…”. How is “no evidence or prior vaccination defined”? It does not appear anti-HBs Ab or anti-HBc Ab are being collected as part of blood work listed so is the evidence merely participants reporting no history of HBV vaccination?

- Consider including footnote with estimated viremic prevalence by cite for sample size calculations in Table 4 for ease of review. Page 17 does provide some information about prevalence and recent programmatic successes that impact HCV viremic prevalence; however, a footnote would assist the reader in quickly appreciating why the sample size estimates are so different by country.

- Supplemental Table 3 and Page 20 – consider explicitly stating in the text if citizenship not required for Tanzanian sites.

- Consider providing additional information about accessibility of the HCV RNA diagnostics by site to detail the potential variability in confirmatory assay accessibility and turn-around-time, possibly as a footnote to Supplementary Table 1. Are there specific numbers of labs by region that will receive the confirmatory samples? Are there estimates of turn-around-times for these results by region?

VERSION 1 - AUTHOR RESPONSE

Reviewer 1: Dr. Jessie Torgersen, University of Pennsylvania

1	Page 13 line 31 suggests many LMIC provide HCV antibody tests are offered free-of-charge as part of harm reduction. Are these antibody tests the POCT diagnostics included in the protocol, such as the OraQuick HCV antibody assay? If not, what is the anticipated cost of the OraQuick assay and how are these being provided for the study activities?	The OraQuick POCTs used in this study are not the POCTs currently available in the study settings; the brand of POCT in use by country programs is dependent on annual national procurement via Global Fund. The OraQuick tests are provided to the study sites by the implementing organisation Medecins du Monde at a cost of approximately 10-12 USD per unit. Please see the added clarifying sentence on page 20:<br>"All eligible participants will be offered sofosbuvir (400mg) and velpatasvir (100mg) (SOF/VEL), to be self-administered orally once per day for 12 weeks. All POC antibody tests will be sourced and funded by the study sponsor Médecins du Monde."
2	The protocol is very clear regarding workflow for confirmatory testing and treatment dispensation. How will treatment interruptions be addressed in the protocol? Is there a certain time point of missed treatment after which the participant will not be provided additional fills of sofosbuvir/velpatasvir? Similarly, how will participants with errors in confirmatory testing be managed (i.e., if confirmatory HCV RNA canceled/not completed after initiation of sofosbuvir/velpatasvir)?	Regardless of how many doses a participant misses, they will be provided with the opportunity to receive 12 weeks worth of treatment. If a participant misses DAA doses throughout their treatment period, they may be provided with additional doses at the discretion of the prescribing clinician. If a participant reports missing more than one week consecutively in the first four weeks, they will be advised to continue taking medication until Week 12 and may be provided an additional 4 weeks of treatment. This is at clinician's discretion and can be decided in consultation with Principal Investigator, on case-by-case basis. Paper edits (see pg 24):<br>Visit 3 – Treatment monitoring and dispensing<br>"Participants' self-reported adherence to treatment will be assessed. Any side effects and any adverse events will be documented. In the event of missed doses, additional weeks of treatment may be provided at the clinician's discretion in consultation with the Principal Investigator."<br><br>For participants in Arm 2 who are provided DAA treatment prior to HCV RNA results, we note on Pg 26 under Visit 1:

		"blood samples will be collected for confirmatory laboratory RNA testing and additional investigations."<br>And<br>"If there is an issue with the blood sample itself, the participant will be asked to return to the study site as soon as possible for a follow up blood draw. Only after confirmatory RNA laboratory results will a second bottle of 28 SOF/VEL doses be provided for the participant to continue their treatment course."
3	Table 2 reports different lower end thresholds for FIB-4 to exclude cirrhosis, specifically 1.25 and 1.45, while the text references 1.45 only for the lower threshold. Please revise Table 2 if the 1.25 value is an error. If not an error, please provide additional rationale for using the 1.25 threshold.	Each country involved in implementation operates under different HCV clinical guidelines which outline HCV treatment for cirrhotic patients based on FIB-4 scores. We have chosen to align our country protocols with country Guidelines to ensure continuity with clinical practice. Please see edits to table 2 clarify this (pg. 23-24).
4	Data will be entered into REDCap; however, it was not clear if data be managed centrally or at each individual site. Consider including a statement about use of data management center, if applicable.	Data will be hosted and managed centrally by Burnet in Australia. This has now been reflected in the manuscript (pg. 36):<br>"Data will be entered into and managed in REDCap, hosted and managed by Burnet Institute in Australia."
5	Will QraQuick performance characteristics be evaluated by HIV status? The validation work by Smookler included only 59 people with HIV across the clinic and Madrid cohorts, the majority of whom had CD4 cell count >200 (where these data were reported). Considering the prevalence of HIV is higher in the regions included in this study, the activities proposed may allow for further evaluation of the performance characteristics by HIV status.	Thank you for this suggestion – we will consider an exploratory sub-analysis of OraQuick performance by HIV status, where data is available. We anticipate that we will have data from Armenia and Tanzania to explore this. We have added this to the manuscript (pg 34):<br>"Where data is available, we will also assess test quick performance characteristics by HIV status."

6	Will there be an analysis of treatment completion as the 12-week treatment course prescribed? Page 34, line 32 describes secondary outcomes, but only defines completion of treatment as completing >=8 weeks of sofosbuvir/velpatasvir. As the prescribed sofosbuvir/velpatasvir course of 12 week, consider indicating if competing the entire prescribed course will be part of the analysis.	This study defines treatment completion as follows:<br>Participants will be classified as completing treatment if they self-report that they have taken at least 8 weeks of treatment, with less than 14 days missed doses. Proportion completed treatment will be assessed in an intention-to-treat (ITT) population of those who were eligible to start treatment at time of treatment prescription. The proportion of participants who complete hepatitis C treatment will be compared between Arms.<br>We have amended the manuscript (pg 33) to include a secondary analysis to assess treatment completion as defined by completing the full 12 week treatment schedule.<br>"We will also assess treatment completion based on completion of the full 12 week treatment course."
7	Will there be quantitative/qualitative assessment of uptake of services such as HIV PrEP and/or HBV vaccination? Co-location of care for PWID is key to reduce barriers to and understanding the acceptability and uptake of these related services to prevent other blood-borne viral infections can inform implementation efforts.	Thank you for this suggestion – we agree that co-location of services is key. While an important topic to explore, this falls outside of the scope of our study, which focuses qualitative interviews on participants' experience in the new HCV models of care specifically.<br><br>Clinical quantitative data is not collected on uptake of HIV PrEP. However, data on HBV vaccination, when offered in partnership with this study, will be collected. Secondary analysis on vaccination uptake may be conducted and would be reported in outcome papers.
8	Throughout the text, there is a shift between MAT and OAT; however, only OAT is defined in the abbreviations (page 18, line 36). Consider revising to keep the abbreviations/terminology consistent.	Thank you for identifying this inconsistency. We have revised the paper to refer to OAT when generally describing opioid maintenance treatment facilities, but have retained MAT when specifically referring to the Tanzanian study site. This is because 'Temeke MAT clinic' is the name used to identify the study site and its staff. MAT has been added to the list of abbreviations.
9	The authors focus on the sensitivity and specificity of the	We have updated "Study Outcomes" Pg 28, to reflect this focus on test performance.

	OraQuick assay (page 29, line 24); however, the PPV will be a key measurement, particularly as it will vary by prevalence of HCV viremia. This is a strength of the analysis - consider revising the text to highlight all performance characteristics of OraQuick will be evaluated, not just sensitivity and specificity.	We have also outlined this analysis under 'Assessing Secondary Outcomes' Pg 34.<br>"The test performance will be determined by calculating the sensitivity and specificity of the shortened five-minute read-time result, and the positive predictive values and negative predictive values."
10	Introduction page 12, line 14 (4th sentence): "Globally, people who inject drugs are a key population groups affected by hepatitis C..." – remove the word "groups" as it redundant.	Thank you for identifying this redundancy. It has now been removed.
11	Introduction page 12, line 16 (5th sentence): "Transmission risk in this groups predominantly due to the re-use of injecting equipment is re-used." Consider revising to, "Transmission risk in this group is predominantly due to the re-use of injecting equipment."	Thank you, this has now been revised as suggested.
12	Page 18, line 35: capitalize the name of the hospital Muhimbili National Hospital in the parentheses.	Thank you, this has now been revised as suggested.
13	Page 23, line 35: "those who return a non-reactive HBsAg RDT and have no evidence of prior hepatitis B vaccination will be offered vaccination...". How is "no evidence or prior vaccination defined"? It does not appear anti-HBs Ab or anti-HBc Ab are being collected as part of blood work listed so is the evidence merely participants reporting no history of HBV vaccination?	Evidence of prior vaccination is sourced from available existing participant medical records, when accessible at the study site. This varies per site, as some sites are hospitals with patient records readily available, and other sites are community-based organizations which do not have access to these records. Please see edits to the protocol below to clarify the source of vaccination 'evidence':<br>"In Tanzania and Armenia, those who return a non-reactive HBsAg RDT and have no evidence of prior hepatitis B vaccination will be offered vaccination through the study. Evidence of vaccination is sourced through medical records accessible by study sites."

14	Consider including footnote with estimated viremic prevalence by cite for sample size calculations in Table 4 for ease of review. Page 17 does provide some information about prevalence and recent programmatic successes that impact HCV viremic prevalence; however, a footnote would assist the reader in quickly appreciating why the sample size estimates are so different by country.	Thank you for this suggestion, please see the addition of a footnote outlining the prevalence estimates in table 4 (pg. 30-31).
15	Supplemental Table 3 and Page 20 – consider explicitly stating in the text if citizenship not required for Tanzanian sites.	Thank you for this suggestion, we have edited the manuscript for clarity (pg. 19):<br>"Be a citizen of the country (Armenia & Georgia – citizenship is not an inclusion criteria for Tanzania) "
16	Consider providing additional information about accessibility of the HCV RNA diagnostics by site to detail the potential variability in confirmatory assay accessibility and turn-around-time, possibly as a footnote to Supplementary Table 1. Are there specific numbers of labs by region that will receive the confirmatory samples? Are there estimates of turn-around-times for these results by region?	Thank you for this suggestion – we agree that the accessibility of RNA diagnostics is important contextual information and turn-around-time can impact clinical pathways. However, the variability between sites' access to RNA diagnostics and pathways to receive diagnostics both within the study and outside of this study is substantial and nuanced. As such, we will present this country and site specific information along with research results in outcome papers, particularly when focusing on next steps for implementation.
